# CD8^+^ T cells stimulate Na-Cl co-transporter NCC in distal convoluted tubules leading to salt-sensitive hypertension

**DOI:** 10.1038/ncomms14037

**Published:** 2017-01-09

**Authors:** Yunmeng Liu, Tonya M. Rafferty, Sung W. Rhee, Jessica S. Webber, Li Song, Benjamin Ko, Robert S. Hoover, Beixiang He, Shengyu Mu

**Affiliations:** 1 Department of Pharmacology and Toxicology, University of Arkansas for Medical Sciences, Little Rock, Arkansas 72205, USA; 2 Department of Pathology, University of Arkansas for Medical Sciences, Little Rock, Arkansas 72205, USA; 3 Department of Medicine, University of Chicago, Chicago, Illinois 60637, USA; 4 Department of Medicine, Division of Nephrology, Emory University, Atlanta, Georgia 30322, USA; 5 Research Service Atlanta, Veteran's Administration Medical Center, Decatur, Georgia 30033, USA

## Abstract

Recent studies suggest a role for T lymphocytes in hypertension. However, whether T cells contribute to renal sodium retention and salt-sensitive hypertension is unknown. Here we demonstrate that T cells infiltrate into the kidney of salt-sensitive hypertensive animals. In particular, CD8^+^ T cells directly contact the distal convoluted tubule (DCT) in the kidneys of DOCA-salt mice and CD8^+^ T cell-injected mice, leading to up-regulation of the Na-Cl co-transporter NCC, p-NCC and the development of salt-sensitive hypertension. Co-culture with CD8^+^ T cells upregulates NCC in mouse DCT cells via ROS-induced activation of Src kinase, up-regulation of the K^+^ channel Kir4.1, and stimulation of the Cl^−^ channel ClC-K. The last event increases chloride efflux, leading to compensatory chloride influx via NCC activation at the cost of increasing sodium retention. Collectively, these findings provide a mechanism for adaptive immunity involvement in the kidney defect in sodium handling and the pathogenesis of salt-sensitive hypertension.

## Abstract (web summary)

T cells contribute to development of high blood pressure but their role in salt-sensitive hypertension is less clear. Liu *et al*. show that CD8^+^ T cells upregulate and activate Na-Cl co-transporter NCC in distal convoluted tubules via direct cell-cell contact and ROS-Src activation, leading to Na^+^ retention and salt-sensitive hypertension.

Hypertension is a major public health problem worldwide with a high prevalence in populations with high dietary salt intake^1^,^2^. It is well established that the kidney plays a key role in the pathogenesis of essential hypertension^3^,^4^,^5^,^6^. A breakthrough in our understanding linking salt intake and kidney function to the pathogenesis of salt-sensitive hypertension was provided by Guyton and other investigators, who proposed that a physiologic defect in the kidney impairs blood pressure-induced sodium excretion, thus leading to salt-sensitive hypertension^7^,^8^,^9^. The thiazide-sensitive sodium-chloride-co-transporter (NCC), which is mainly expressed in distal convoluted tubules (DCT), plays a major role in sodium handling in the distal nephron^10^,^11^,^12^. Genetic mutations of NCC or its regulatory factors lead to salt wasting or salt-sensitive effects on blood pressure regulation^13^,^14^,^15^,^16^. Inactivating mutations of NCC lead to Gitelman's syndrome with hypotension^13^,^14^, whereas over-activation of NCC by mutations of its with-no-lysine (WNK) regulators results in Gordon syndrome, exhibiting hypertension^15^,^16^. Recent studies demonstrate that intracellular chloride importantly regulates NCC and the sodium-potassium-chloride co-transporter (NKCC) by affecting their regulatory pathways, including auto-phosphorylation of WNKs and their interaction with Ste20-related proline–alanine-rich kinase (SPAK)^17^,^18^,^19^. However, which chloride channel or transporter in DCT cells is responsible for alterations in intracellular chloride remains unclear.

The renal tubular chloride channel ClC-K, which is expressed throughout the distal nephron and located on the basolateral membrane, plays a pivotal role in chloride reabsorption^20^,^21^. There are two known homologues of this channel, ClC-K1 and ClC-K2. The distribution pattern of each ClC-K variant in the distal nephron is uncertain because of the lack of specific antibodies, but they both require association with their beta subunit-barttin (Bsnd) to be functional^22^. Loss-of-function mutations of ClC-K or Bsnd in the thick ascending limb of the loop of Henle are responsible for classic Bartter syndrome (type III & IV) accompanied by salt wasting, hypokalemic alkalosis, and hypercalciuria^23^,^24^. Although direct evidence of ClC-K regulating NCC is missing, patients carrying ClC-K mutations demonstrate Gitelman's syndrome^25^,^26^ leading us to speculate that the NCC in DCT segments is affected by the function of ClC-K. Recent studies suggest that changes in plasma K^+^ concentration and the basolateral K^+^ channel Kir4.1, a known downstream target of Src kinases, may play important roles in regulating ClC-K, consequently affecting NCC expression and activation^27^,^28^,^29^,^30^. However, direct evidence linking the regulation of Kir4.1 and the pathogenesis of salt-sensitive hypertension is missing.

A role for the immune system in hypertension was proposed in the 1960s (refs 31, 32) and is supported by the following observations: Immuno-compromised nude mice are less able to maintain hypertension in response to DOCA-salt treatment compared with immuno-competent mice^33^; thymus transplantation from WKY rats to SHR lowers blood pressure in SHR^34^; and dysfunction of immune cells caused by Rag-1 knockout/mutation or the immunosuppressant mycophenolate-mofetil blunts the elevated blood pressure in DOCA-salt treated animals or Dahl salt-sensitive rats^35^,^36^,^37^. More recently, landmark studies by Harrison and colleagues^35^ provide evidence for a pathophysiological role of T cells in the development of hypertension. Adoptive transfer of T cells to Rag1 knockout mice restored elevation of blood pressure caused by Angiotensin II (AngII) infusion^35^. These investigators also demonstrated the relative importance of T cell sub-types in the development of hypertension: adoptive transfer of CD8^+^ T cells, but not CD4^+^ T cells, promoted the development of hypertension^38^. Further confirmation included the observation that knockout of CD8 prevented hypertension in AngII or DOCA-salt treated mice^39^. Although growing evidence supports a role for T cells in the pathogenesis of hypertension, whether T cells contribute to the kidney defect in sodium handling in salt-sensitive hypertension is unclear. Interestingly, recent studies demonstrate that IFNγ and IL17a are involved in AngII-induced NCC up-regulation and activation in kidney^40^,^41^. However, whether inflammatory cytokines play a bridging role between T cells and sodium retention remains to be tested.

In this study, we hypothesized a novel pathophysiologic mechanism of salt retention in hypertension: that T cells in the kidney stimulate NCC in DCTs, leading to sodium retention and salt-sensitive hypertension. We found that CD8^+^ T cells stimulate NCC in mouse DCTs by upregulating the potassium channel Kir4.1 and subsequently the chloride channel ClC-K on the plasma membrane, thereby decreasing intracellular chloride. The last event leads to NCC activation, sodium retention and the development of salt-sensitive hypertension. Furthermore, we found that CD8^+^ T cell-mediated NCC up-regulation in DCT cells requires direct cell-cell interaction-induced ROS-Src activation.

## Results

### CD8^+^ T cells upregulate NCC in salt-sensitive hypertension

**Figure 1:**
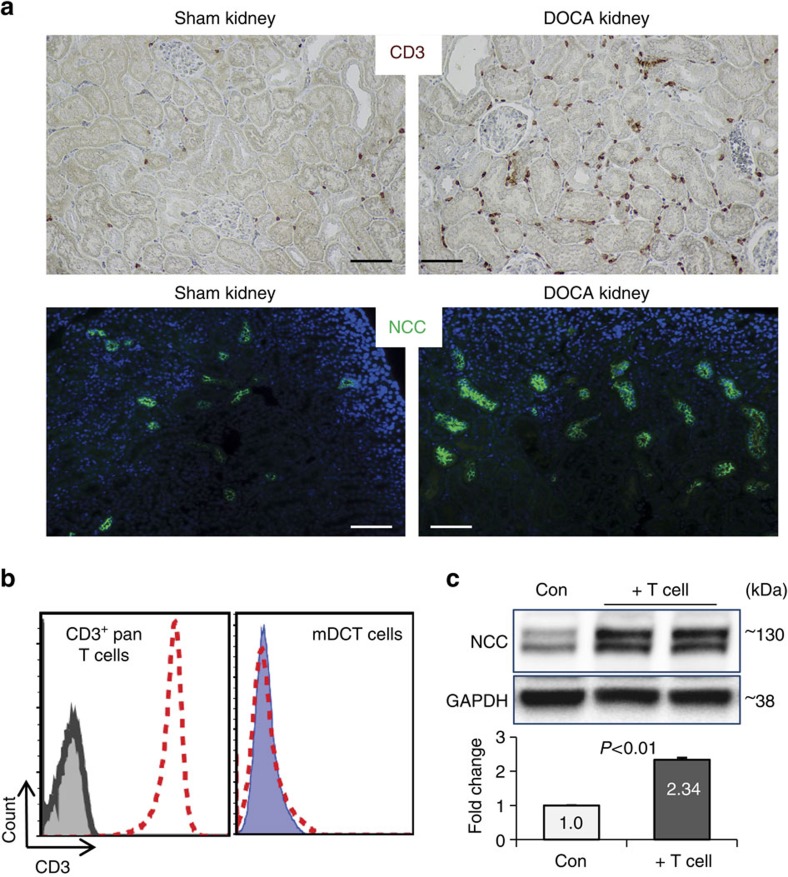
T cells accumulate in the kidney of DOCA mice and are associated with NCC up-regulation. (**a**) Immunostaining of pan T cell marker CD3 (brown, upper panels, scale 50 μm) or NCC (green, lower panels, scale 100 μm) on Sham or DOCA-salt treated mouse kidney sections. Nuclei were stained by hematoxyline (upper panels) or DAPI (lower panels). Data are representative of *n*=8 images in each group. (**b**) Left panel, isolated mouse splenic pan T cells were stained by CD3 antibody. Flow cytometry confirmed all cells were CD3^+^ T cells. Cells in grey closed area are T cells without CD3 staining as negative control; cells in red dashed line open area are T cells stained by CD3 antibody, indicating CD3^+^ T cells. Right panel, effects of using magnetic beads to remove T cells from the mDCT-T cell co-culture. Control mDCTs (before co-culture, blue closed area) and after co-culture mDCTs (after removal of T cells by using magnetic beads, red dashed line open area) were stained with CD3 antibody and analysed by flow cytometer. Data are representative of three independent tests. (**c**) Western blot analysis of NCC expression in mDCT cells with (+T cell) or without (Con) mouse splenic T cell-treatment. Two bands were detected for NCC, reflective of mature (upper) and immature (lower) forms (see methods). Quantitative western blot data were normalized using GAPDH as a loading control. *n*=4–5 in each group. Data are means±s.e. *P*<0.01 (t-test).

DOCA-salt treated hypertensive mice (DOCA mice) and Dahl salt-sensitive rats are two classic animal models of salt-sensitive hypertension^42^. We verified their elevated blood pressure via direct measurement (Supplementary Fig. 1). Staining their kidneys with the T cell specific marker CD3 confirmed the earlier finding^39^,^43^ that massive T cell infiltration occurs in salt-sensitive hypertensive animals compared with control animals (Fig. 1a upper panel; Supplementary Fig. 2). Up-regulation of NCC in the kidney of DOCA mice was shown by immunofluorescent staining with a specific NCC antibody (Fig. 1a, lower panel). To determine whether T cells are involved in the up-regulation of NCC in DCT cells, we extracted pan T cells (CD3^+^, Fig. 1b left) from spleens of C57B6 mice and co-cultured them with mDCT15 cells (mDCTs), an established cell model of mouse DCT^44^. After removal of the T cells, mDCTs (Fig. 1b right) analysed by immunoblot showed higher NCC expression in T cell-treated mDCTs compared with untreated control cells (Fig. 1c), suggesting that T cells trigger increased NCC expression in mDCTs.

**Figure 2:**
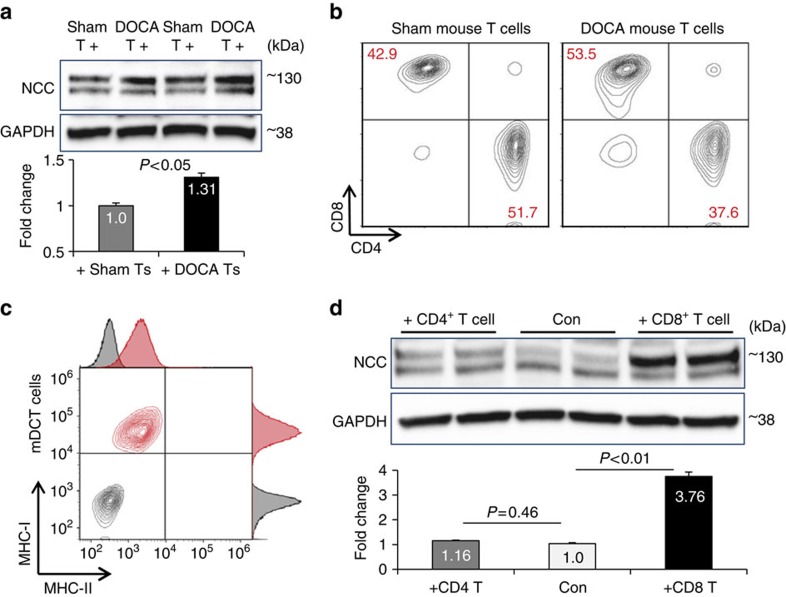
CD8 subtype T cells are involved in the up-regulation of NCC. (**a**) Expression of NCC in mDCT cells treated with T cells from spleens of Sham (Sham T+) or DOCA (DOCA T+) mice. After removal of all T cells, whole cell lysates were used for western blot. GAPDH was used as loading control. *n*=4 in each group. Data are means±s.e. *P*<0.05 (*t*-test). (**b**) Pan T cells isolated from spleens of Sham or DOCA mice were analysed for the proportion (%, red numbers) of CD4 and CD8 subtype by flow cytometry. Data are representative of three independent experiments. (**c**) mDCT cells with (red) or without (grey, negative control for auto-fluorescence) double staining using MHC-I (*Y* axis) & MHC-II (*X* axis) antibodies were analysed by flow cytometry. Data are representative of four independent experiments. (**d**) Western blot analysis of NCC expression in mDCT cells without or with CD4^+^ or CD8^+^ mouse splenic T cell-treatment. Quantitative western blot data were normalized using GAPDH as a loading control. *n*=4–5 in each group. Data are means±s.e. *P*=0.46 Con versus +CD4T; *P*<0.01 Con versus +CD8T (ANOVA).

Next, we compared treatment of mDCTs with T cells obtained from spleens of Sham mice or DOCA mice. T cells from DOCA mice stimulated more NCC expression than those from Sham mice (Fig. 2a). T cell subtype analysis by flow cytometry identified a higher proportion of CD8^+^ T cells and a lower proportion of CD4^+^ cells in the T lymphocytes (CD3^+^) isolated from DOCA mice compared with Sham mice (Fig. 2b; Supplementary Fig. 3). This observation led us to speculate that the CD8^+^ subtype of T cell is more important in stimulating NCC in mDCTs. To test this hypothesis, we evaluated the expression of the CD4/CD8-specific antigen ligand MHC-II/MHC-I on the surface of mDCT cells. Flow cytometry revealed abundant expression of CD8-specific antigen MHC-I on the mDCT cell surface with minor expression of CD4 specific antigen MHC-II (Fig. 2c). We then co-cultured mDCTs with mouse CD4^+^ T cells and CD8^+^ T cells isolated from spleens using negative selection (Supplementary Fig. 4) to test their effects on regulating NCC. Co-culture with CD8^+^ T cells greatly upregulated NCC expression in mDCT cells, whereas the effect from CD4^+^ T cells was modest (Fig. 2d).

**Figure 3:**
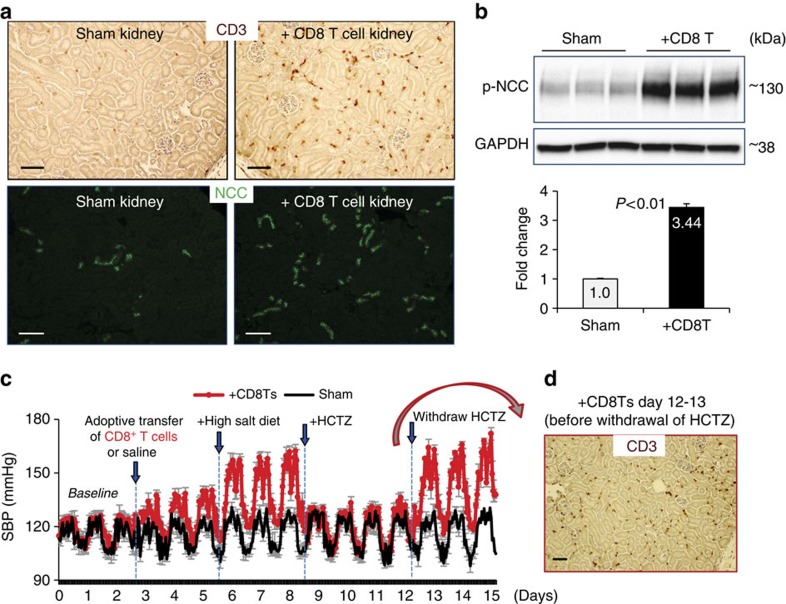
Adoptive transfer of CD8^+^ T cells to mice results in salt-sensitive hypertension. (**a**) Immunostaining of pan T cell marker CD3 (brown, upper level, scale 50 μm) or NCC (green, lower level, Scale, 100 μm) in kidneys from sham mice or mice receiving CD8^+^ T cell-adoptive transfer (+CD8 T cell). Data are representative of *n*=10 images in each group. (**b**) Western blot analysis of p-NCC expression in the kidneys of sham mice and mice receiving CD8^+^ T cell-adoptive transfer (+CD8 T). Quantitative western blot data were normalized using GAPDH as a loading control. *n*=5–6 in each group. Data are means±s.e. *P*<0.01 (*t*-test). (**c**) Averaged radio-biotelemetry recording of systolic blood pressure in 5 mice with saline injection (sham, black line) and 10 mice receiving adoptive transfer of CD8^+^ T cells (+CD8Ts, red line). All mice were given high-salt diet and HCTZ drinking water as indicated. Blood pressure was recorded hourly. Data are means±s.e. (**d**) At day 13 (last day of HCTZ treatment) 4 mice from +CD8 T group were removed from the blood pressure recording and their kidneys were used for immunostaining for CD3. Scale, 50 μm.

To verify our *in vitro* result, mouse CD8^+^ T cells were infused into mice via tail vein injection. Tracking these exogenous CD8^+^ T cells using fluorescent dye revealed their infiltration into the kidneys and spleens but not the hearts of the recipient mice (Supplementary Fig. 5). Five days after adoptive transfer of CD8^+^ T cells, massive T cell infiltration and up-regulation of the NCC were detected in the mouse kidneys by immuno-(fluorescent)-staining (Fig. 3a), similar to the result we observed in DOCA mice (Fig. 1a). Moreover, phosphorylated NCC (p-NCC), an activated form of NCC, also was up-regulated in the kidneys of mice infused with CD8^+^ T cells (Fig. 3b). Next, blood pressure was measured by radio-biotelemetry to evaluate whether CD8^+^ T cell-induced up-regulation of the NCC in kidney is associated with salt-sensitive hypertension. The mice receiving adoptive transfer of CD8^+^ T cells (Fig. 3c, red) showed slightly higher blood pressure (119±2.4/132±2.1 mmHg, sys-BP day/night) compared with their baseline (113±1.9/122±1.3 mmHg). However, remarkably elevated blood pressure was observed in these mice after their diet was switched from regular chow to 8% high-salt diet (128±2.8/152±2.6 mmHg). Furthermore, the NCC blocker hydrochlorothiazide (HCTZ) effectively lowered blood pressure in these mice (111±2.8/125±1.8 mmHg), suggesting a critical role for NCC in the development of salt-sensitive hypertension in mice that received adoptive transfer of CD8^+^ T cells. In contrast, blood pressure in sham mice was unaffected by injection of saline, high-salt diet, or ±HCTZ treatment (Fig. 3c, black). It is noteworthy that, because T cell infiltration in the kidney was not diminished by treatment with HCTZ (Fig. 3d), removal of HCTZ regenerated hypertension in mice receiving adoptive transfer of CD8^+^ T cells (Fig. 3c, red, 129±4.0/155±3.3 mmHg), suggesting an upstream role for renal CD8^+^ T cells in NCC-blood pressure regulation. Our *in vitro* and *in vivo* data suggest that CD8^+^ T cells play a major role in up-regulation and activation of NCC in DCTs and that upregulated NCC in kidneys contributes to the development of salt-sensitive hypertension in CD8^+^ T cell-infused mice.

### CD8^+^ T-DCT direct contact is required for upregulating NCC

**Figure 4:**
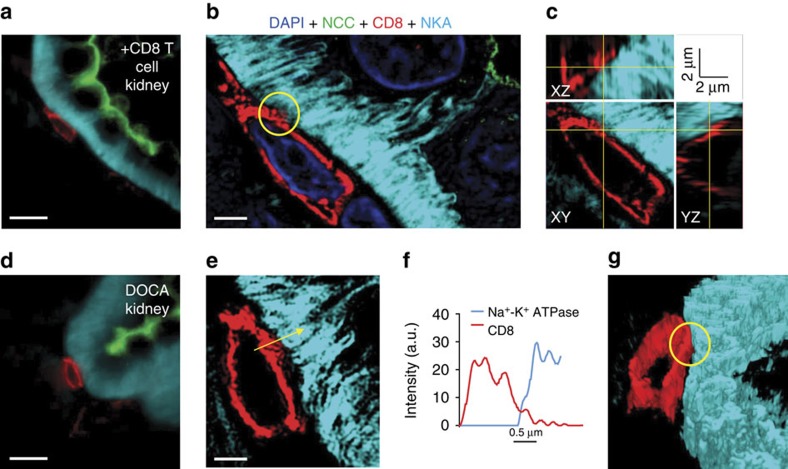
CD8^+^ T cells form close contacts with distal convoluted tubules in mouse kidney sections. (**a**) A wide-field image of a kidney section from a mouse injected with CD8^+^ T cells. Scale 10 μm. Antibodies against NCC (green), CD8 (red), and Na^+^-K^+^-ATPase-α (NKA, imaged far red and pseudo-colored cyan for visual clarity) were used. (**b**) The same CD8^+^ T cell from (**a**) processed with 3D structured illumination microscopy (3D-SIM). Scale, 2 μm. Four colour channel images were overlaid: DAPI (blue), NCC (green), CD8 (red), and NKA (cyan). The yellow circle denotes the putative contact point. (**c**) Orthogonal sections of the 3D image (XZ, XY, YZ) of the same cell. Yellow lines on each section correspond to the position of other orthogonal sections centred around the contact point. (**d**) A wide-field image of a kidney section from a DOCA-salt mouse. Identically stained as in **a**. Scale 10 μm. (**e**) 3D-SIM processed image of (**d**). Scale 2 μm. Yellow arrow represents the location and direction of intensity profile. (**f**) Fluorescence intensity profile of CD8 (red) and NKA (cyan) along the yellow arrow in **e**. (**g**) Three-dimensional surface rendering of the image shown in **e**. The yellow circle denotes the putative contact point. Data are representative of 11 images from +CD8 T cell group and 13 images from DOCA group.

Using double fluorescent staining of NCC and CD8 to localize NCC-positive DCTs and CD8^+^ T cells, we found numerous DCT segments with high NCC expression surrounded by CD8^+^ T cells in kidneys of both DOCA mice and mice receiving CD8^+^ T cells (Supplementary Fig. 6). This finding led us to consider the possibility that direct cell–cell contact may be involved in the interaction between CD8^+^ T cells and DCT cells. To test this hypothesis, the basolateral membrane of DCTs was stained for Na-K-ATPase-α, and super-resolution 3D-SIM (three dimension structured illumination microscopy) was used to examine whether CD8^+^ T cells directly touch the basolateral side of NCC-positive DCT cells *in vivo* (Fig. 4). Raw images (Fig. 4a,d) spanning ∼4.5 μm Z-stacks were processed to yield 3D-SIM images (Fig. 4b,e) and orthogonal sections (Fig. 4c), linear intensity profiles (Fig. 4f) and 3D-surface-rendering images (Fig. 4g) were examined. Within the resolution limit^45^ of 3D-SIM, protrusions from CD8^+^ T cells (red) appear to make contact with NCC (green)-positive DCTs on the basolateral membrane (cyan) in kidneys from both DOCA mice (Fig. 4a–c) and mice receiving CD8^+^ T cell adoptive transfer (Fig. 4d–g).

**Figure 5:**
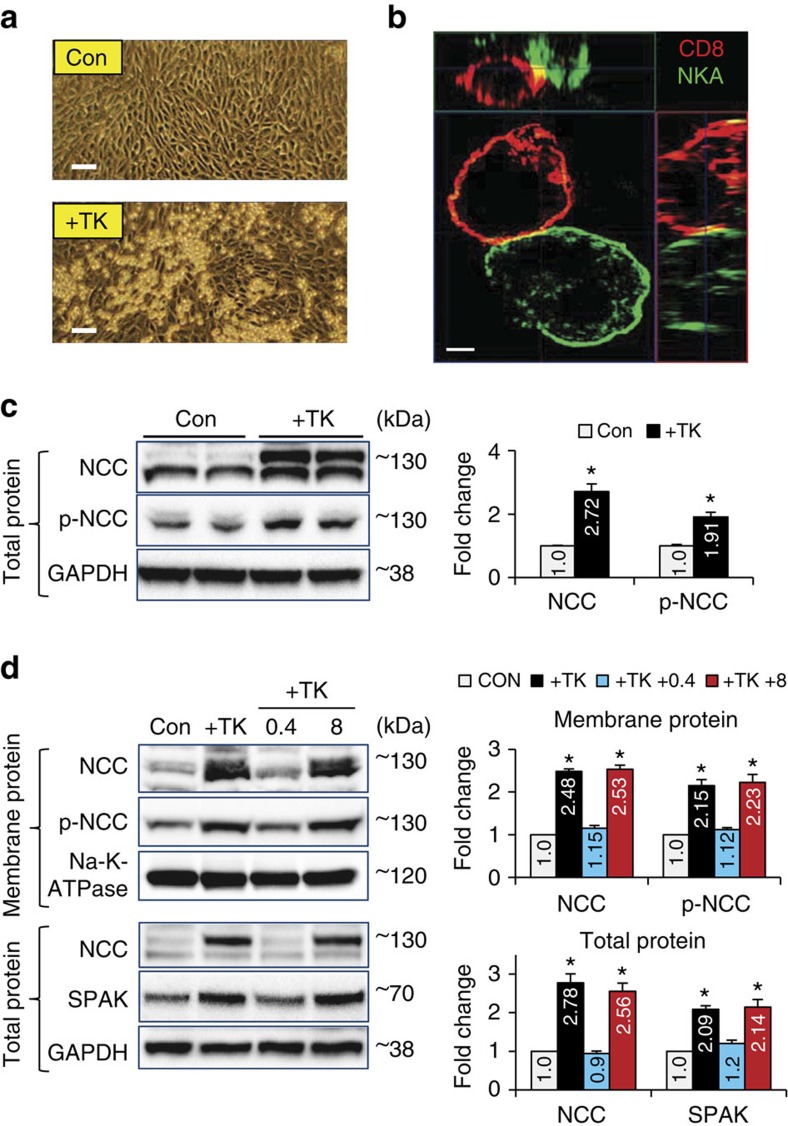
TKs (a CD8^+^ T cell line) upregulate NCC in mDCTs via direct cell-cell contact. (**a**) Image of TK cells adherent to mDCTs. Scale, 40 μm. After overnight co-culture, cells were washed with PBS × 2. Data are representative of *n*=10 images in each group. (**b**) Orthogonal section of the 3D image (XZ, XY, YZ) of individual cell-cell contact of TK (CD8, red) and mDCT (Na-K-ATPase, NKA, green) cells in co-culture for 4 h. Scale, 2 μm. Data are representative of *n*>15 images. All images were captured and processed with super-resolution 3D-SIM microscopy. (**c**) Total protein expression of NCC and p-NCC in mDCTs with or without TK co-culture. GAPDH was used as a loading control. *n*=4–6 in each group. Data are means±s.e. *P<0.01 versus Control (*t*-test) (**d**) Upper, effects of 0.4 and 8 μm transwells on TK-mediated up-regulation of NCC and p-NCC in mDCT membrane protein. Lower, effects of transwells on TK-mediated up-regulation of NCC and full length SPAK expression in mDCT total lysate. All groups with or without transwells used the same culture conditions. Na-K-ATPase or GAPDH were used as loading controls of membrane protein or total protein, respectively. *n*=4 in each group. Data are means±s.e. *P<0.01 versus Control (ANOVA).

To determine whether this direct cell-cell contact is critical for the interaction between CD8^+^ T cells and DCTs, and further elucidate the mechanism of CD8^+^ T cell-induced up-regulation of NCC in mDCTs, we co-cultured the mouse CD8^+^ T lymphocyte line TK-1 cells (TKs) (Supplementary Fig. 7) with mDCTs. Consistent with our *in vivo* observation, many TKs adhered to mDCTs (Fig. 5a) and individual cell-cell contacts were detected by super-resolution-microscopy (Fig. 5b; Supplementary Fig. 8). After co-culture, mDCT-adherent TKs were removed by CD8 specific magnetic beads and TK-free mDCTs were further analysed (Supplementary Fig. 9a). Western blots showed that the abundance of total NCC and p-NCC increased in TK-treated mDCTs compared with control (Fig. 5c), although the corresponding mRNA change was minor (Supplementary Fig. 9b). TK-mediated up-regulation of NCC and p-NCC on the cell membrane of mDCTs was confirmed by western blot of isolated cell membrane proteins (Supplementary Fig. 9c).

To determine whether TK-induced up-regulation of NCC is because of direct cell-cell contact or cytokines or chemokines released from the T cells, transwell co-culture inserts with different membrane pore size (0.4 and 8 μm) were used to separate TKs and mDCTs. The 0.4 μm transwell should allow exchange of secreted, soluble cytokines/chemokines but prevent direct cell-cell contact, whereas the 8 μm transwell allows free trans-membrane movement of both soluble factors and TK cells, since the TKs are only ∼7–9 μm in diameter (Supplementary Fig. 10a,b). Western blots revealed that TKs increase membrane abundance of NCC and p-NCC only in mDCTs without transwell or with 8 μm-transwell that allows TKs to directly touch mDCTs, but not in the groups of control cells or with the 0.4 μm-transwell that prohibits direct contact between the two cell types (Fig. 5d upper panels). In total protein lysates, NCC and its major regulator SPAK (refs 46, 47) only up-regulated consistently in the mDCTs that directly contacted TKs. Indirect co-culture did not stimulate the expression of NCC or SPAK (Fig. 5d lower panels). Moreover, specific blocking antibodies used to neutralize cytokines IL17a and IFNγ in the co-culture failed to prevent TK-induced up-regulation of SPAK and NCC in mDCTs (Supplementary Fig. 11), which further confirmed these cytokines do not direct mediate TK-induced NCC up-regulation in mDCTs. These results indicate that CD8^+^ T cell-mediated NCC up-regulation and activation in DCTs relies on direct cell-cell interaction and not on soluble factors.

### The upregulated NCC mediates more sodium uptake in DCTs

**Figure 6:**
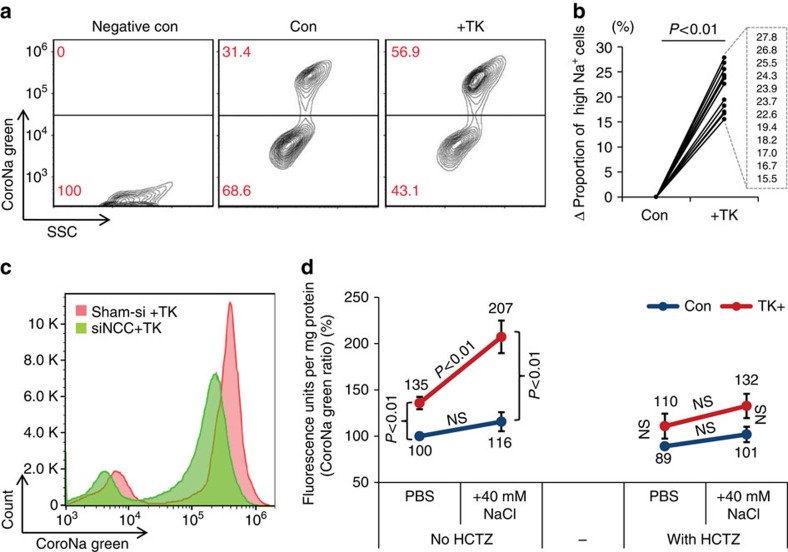
TKs induce more mDCTs to exhibit high sodium uptake ability via NCC. (**a**) Flow cytometry study using intracellular sodium indicator CoroNa Green. All groups were in presence of ouabain (500 μM), bumetanide (100 μM) and amiloride (100 μM). Negative control without CoroNa Green represents auto-fluorescence of mDCT cells. *x*-axis, side scatter (SSC); *y*-axis, CoroNa Green fluorescence. Data are representative of twelve independent experiments. (**b**) Change (Δ) in the proportion of cells with elevated sodium. *n*=12 groups. *P*<0.01 (*t*-test). (**c**) Effects of NCC knockdown by siRNA (siNCC) on TK-induced sodium uptake in mDCTs. *x*-axis, CoroNa Green fluorescence, *y*-axis, cell number count. Data are representative of four independent experiments. (**d**) Ratio quantification of TK-induced Na^+^ uptake in mDCTs with or without NCC inhibitor HCTZ (200 μM) in regular PBS incubation (PBS) or in incubation with additional salt (+40 mM NaCl). *n*=6–8 in each group. Data are means±s.e. *P*<0.01 (ANOVA). NS, not significant.

We next performed functional studies using a membrane-permeable fluorescent intracellular sodium indicator CoroNa Green AM (CoroNa) to evaluate NCC-mediated sodium retention in mDCTs. After overnight treatment with or without TKs, mDCTs were incubated in PBS containing the Na-K-ATPase inhibitor ouabain, NKCC blocker bumetanide and ENaC inhibitor amiloride, followed by loading CoroNa in the presence of the same blockers. Compared with the negative-control cells without sodium indicator loading, CoroNa-loaded mDCTs demonstrated two major populations: lower sodium-containing and higher sodium-containing mDCTs (Fig. 6a). Notably, TK-treated mDCTs exhibited a higher proportion of cells with high sodium retention compared with control cells (Fig. 6a,b), suggesting TKs stimulate more mDCTs to exhibit elevated sodium-reabsorbing ability, conceivably via enhanced NCC activation. To confirm the role of NCC in TK-mediated sodium retention in mDCTs, we used siRNA to knockdown NCC in mDCTs before treatment with TKs (Supplementary Fig. 12a). NCC knockdown by siRNA decreased the proportion of high sodium-containing mDCTs induced by TKs (Fig. 6c & Supplementary Fig. 12b). Moreover, knockdown of NCC in mDCTs shifted the entire fluorescence curve to the left (Fig. 6c), indicating decreased sodium retention in the whole cell population.

To semi-quantitatively assess the sodium absorbed into mDCTs by activated NCC, the NCC-specific inhibitor HCTZ was added to the other sodium transporter blockers mentioned above. An additional 40 mM NaCl was added to the incubation PBS to mimic the high-salt environment found in the renal interstitium after high-salt-intake^48^. CoroNa-loaded mDCTs were lysed in sodium free buffer and fluorescence was measured by fluorometry. In normal salt condition, mDCTs treated with TKs demonstrated higher sodium content than control cells (Fig. 6d), consistent with the flow cytometry result (Fig. 6a). The additional high-salt induced a rise in sodium content in mDCTs that was minimal in control cells, but dramatic in TK-treated mDCTs (Fig. 6d). Moreover, this effect was abolished by inhibiting NCC with HCTZ. After HCTZ administration, the sodium content in TK-treated mDCTs was diminished to a level similar to control cells (Fig. 6d). These results suggest that TK-induced up-regulation and activation of NCC contribute to the sodium retention in mDCTs.

### Chloride efflux contributes to NCC up-regulation in DCTs

**Figure 7:**
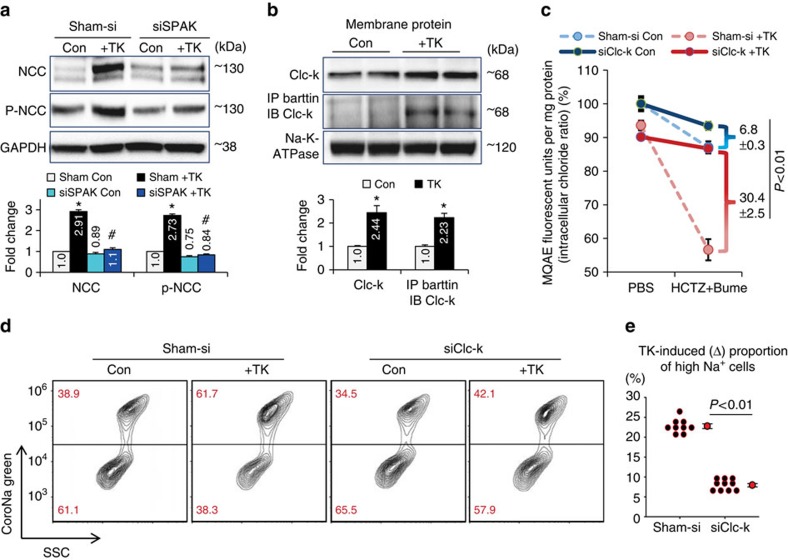
Effects of SPAK or ClC-K knockdown on NCC and intracellular chloride. (**a**) SPAK knockdown using siRNA (siSPAK) prevented the up-regulation of NCC & p-NCC in TK-treated mDCTs. Total lysate protein loading was normalized by GAPDH. *n*=4–6 in each group. Data are means±s.e. *P<0.01 versus Control; ^#^*P*<0.01 versus sham+TK (ANOVA). (**b**) Membrane expression of ClC-K was detected by western blot. The binding of membrane ClC-K to its subunit barttin was detected by immunoprecipitation (IP) of barttin and immunoblot (IB) of ClC-K. Membrane protein loading for both western blot and IP/IB were normalized to Na-K-ATPase (see Methods). *n*=4–6 in each group. Data are means±s.e. *P<0.01 versus Control (*t*-test). (**c**) ClC-K knockdown using siRNAs (siClC-K) prevented the reduction of intracellular chloride in TK-treated mDCTs. Intracellular chloride measured with MQAE. The difference between PBS and HCTZ+Bume of each line reflects NCC/NKCC compensated chloride efflux in each group of mDCTs. TK-induced chloride efflux (in presence of HCTZ+Bume) was compared between the groups without siClC-K (Sham-si, Dashed lines) and with siClC-K (solid lines). Data are means±s.e. *n*=6–10 in each group. *P*<0.01 (*t*-test). (**d**) NCC-mediated sodium uptake in control or TK-treated mDCTs with or without ClC-K knockdown was measured using the same method as in Fig. 5. Data are representative of nine (sham-si) to ten (siClC-K) experiments. (**e**) TK-induced change (Δ) in the proportion of cells with elevated sodium. *n*=9–10 groups. Data are means±s.e. *P*<0.01 (*t*-test).

Recent reports have shown intracellular chloride concentration is an important factor affecting NCC/NKCC regulatory pathways including SPAK (refs 17, 18, 19), which led us to investigate whether TK-induced NCC up-regulation is mediated by the intracellular chloride-SPAK pathway. Indeed, knockdown of SPAK using siRNA (Supplementary Fig. 13a) prevented TK-induced NCC/p-NCC up-regulation in mDCTs (Fig. 7a). To measure intracellular chloride, the membrane-permeable fluorescent chloride indicator MQAE was loaded into chloride-depleted mDCTs, followed by incubation in PBS with or without NCC/NKCC blockers. Without blockers, the treatment with TKs only slightly decreased intracellular chloride concentration in mDCTs (Supplementary Fig. 13b). Although TKs did not significantly upregulate NKCC in mDCTs (Supplementary Fig. 13c), blocking NCC- or NKCC-mediated chloride influx with specific inhibitors HCTZ or bumetanide (Bume) each mildly decreased intracellular chloride in TK-treated mDCTs (Supplementary Fig. 13b). As expected, combined blockade with both HCTZ and Bume remarkably decreased intracellular chloride in TK-treated mDCTs compared with control cells (Supplementary Fig. 13b), indicating a much higher chloride efflux in the mDCTs with TK treatment than control. In the absence of blockers, this chloride efflux was largely compensated by NCC/NKCC-mediated chloride influx.

A previous patch-clamp study identified ClC-K as a major basolateral membrane chloride channel in DCTs (ref. 49). To clarify whether it is responsible for the chloride efflux in TK-treated mDCTs, we performed membrane extraction and observed more ClC-K expressed on the cell membrane in TK-treated cells in western blots (Fig. 7b). Moreover, using immunoprecipitation, we detected more Barttin-ClC-K association on the plasma membrane of TK-treated mDCTs (Fig. 7b; Supplementary Fig. 14a), indicating activation of the increased surface ClC-K. These results suggested that TK-treatment induces higher mDCT membrane expression and activation of ClC-K, which may be responsible for the higher chloride efflux from mDCTs.

To confirm the role of ClC-K in TK-mediated chloride efflux from mDCTs and its relation to TK-NCC-mediated sodium retention, we used both ClC-K1 and ClC-K2 siRNAs to knock down ClC-K (Supplementary Fig. 14b,c). ClC-K knockdown by siRNAs prevented the reduction in intracellular chloride in both control and TK-treated mDCTs with HCTZ/Bume blockade. Importantly, the effect was greater in TK-treated mDCTs than in control cells (Fig. 7c). Notably, after ClC-K knockdown, the slope of the (HCTZ+Bume)-induced sharp decrease of intracellular chloride in TK-treated mDCTs was similar to control cells (Fig. 7c), which confirmed that ClC-K critically modulates chloride efflux from mDCTs. NCC-mediated sodium retention in mDCTs was measured by flow cytometry using CoroNa Green as described earlier. Without knockdown of ClC-K, TK-treatment resulted in a greater proportion of high sodium containing mDCTs compared with control cells (Fig. 7d,e, Sham-si), consistent with our finding in Fig. 6. However, this effect was diminished in the mDCTs after knockdown of ClC-K (Fig. 7d,e, siClC-K). These results suggest that TK-treatment increases mDCT membrane expression and activation of ClC-K, and moreover, it decreases intracellular chloride, thereby controlling SPAK-NCC-mediated sodium reabsorption in TK-treated mDCTs.

### CD8^+^ T cells activate the Kir4.1-ClC-K pathway in DCTs

**Figure 8:**
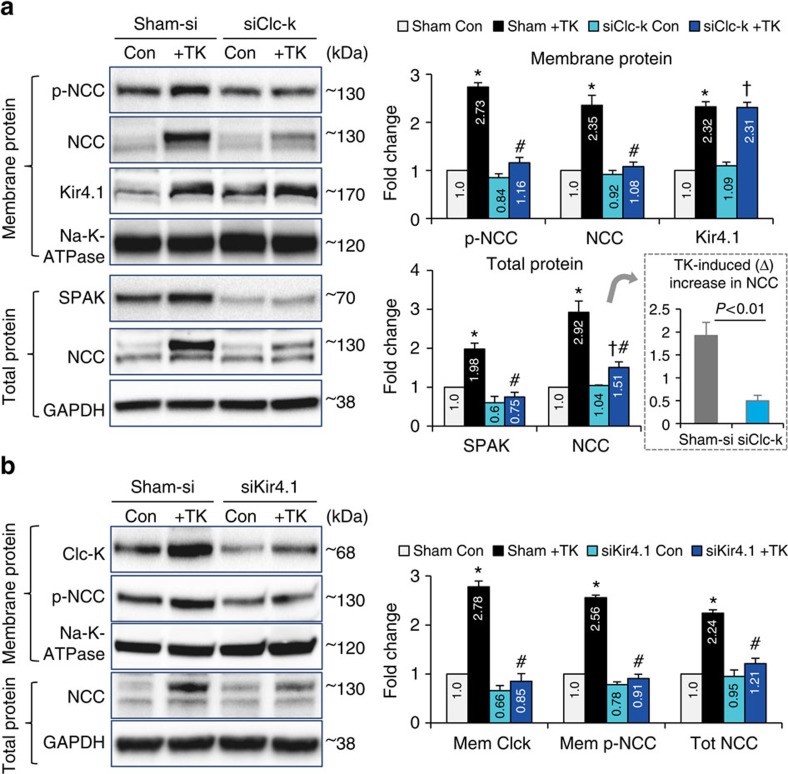
Effects of ClC-K or Kir4.1 knockdown on TK-mediated NCC-regulating pathway in mDCTs. (**a**) In membrane proteins (upper), ClC-K knockdown prevented TK-induced membrane expression of p-NCC and NCC but did not affect the up-regulation of Kir4.1. *n*=4–6 in each group. Data are means±s.e. *P<0.01 versus sham control; ^#^*P*<0.01 versus sham+TK; ^†^*P*<0.01 versus siClC-K Con (ANOVA). In total protein lysates (lower), TK-mediated up-regulations of SPAK and NCC were also diminished by siClC-K administration. *n*=4–5 in each group. Data are means±s.e. *P<0.01 versus sham control; ^#^*P*<0.01 versus sham+TK (ANOVA). TK-induced (Δ) increase in total NCC expression was compared between sham-si and siClC-K groups (Dashed lined area). *n*=4–5 in each group. Data are means±s.e. *P*<0.01 versus sham-si (*t*-test). Na-K-ATPase or GAPDH were used as loading controls of membrane protein or total protein, respectively. (**b**) Kir4.1 knockdown prevented TK-induced up-regulation of ClC-K and p-NCC in mDCT cell membrane (upper) and NCC in total lysate (lower). Na-K-ATPase or GAPDH were used as loading controls for membrane protein or total protein, respectively. *n*=4–6 in each group. *P<0.01 versus Control; ^#^*P*<0.01 versus sham+TK (ANOVA).

To further investigate the role of ClC-K in the pathway of TK-induced NCC up-regulation, western blots were performed using membrane protein and total protein from TK-treated/-untreated mDCTs with or without ClC-K knockdown. Not surprisingly, ClC-K knockdown prevented TK-induced up-regulation of ClC-K on the cell membrane (Supplementary Fig. 14d). Moreover, TK-induced increases of NCC and p-NCC in the cell membrane and SPAK and NCC in whole cell lysate were abolished by siClC-K (Fig. 8a). These data suggest that ClC-K is an important upstream regulator of NCC activation and expression in mDCTs. Zhang *et al*.^30^ recently reported that the DCT basolateral potassium channel Kir4.1 provides the driving force for chloride efflux. Knockout of Kir4.1 decreased chloride conductance and impaired NCC expression in mouse DCT1 (ref. 30). In agreement, our findings imply that TKs up-regulate mDCT membrane expression of Kir4.1 and this response is not impaired by ClC-K knockdown (Fig. 8a), suggesting that Kir4.1 is an up-stream regulator of ClC-K in DCTs.

In further confirmation, knockdown of Kir4.1 by siRNA (Supplementary Fig. 15a) prevented TK-induced up-regulation of Kir4.1, ClC-K and p-NCC on mDCT cell membrane (Fig. 8b; Supplementary Fig. 15b). In addition, the TK-induced increase in total NCC abundance in mDCT total lysate also was abolished by siKir4.1 (Fig. 8b). These data suggest that TK-induced NCC up-regulation and activation occur via up-regulation of the Kir4.1-ClC-K pathway. Recent reports from Terker *et al*.^27^ and Veiras *et al*.^28^ indicated that plasma potassium plays an important role in regulating NCC in DCTs, with Kir4.1 possibly involved^27^. However, we did not observe a decrease in potassium level in plasma of mice receiving CD8^+^T cell-adoptive transfer (Supplementary Fig. 16a, left panel) or in the mDCT-TK co-culture media (Supplementary Fig. 16a, right panel). Moreover, additional potassium in the co-culture environment did not attenuate the TK-induced NCC up-regulation in mDCTs (Supplementary Fig. 16b), confirming that CD8^+^ T cell-induced NCC up-regulation in DCT occurs via a different mechanism than extracellular potassium.

### CD8^+^ T cells stimulate ROS-Src in DCTs to activate Kir4.1

**Figure 9:**
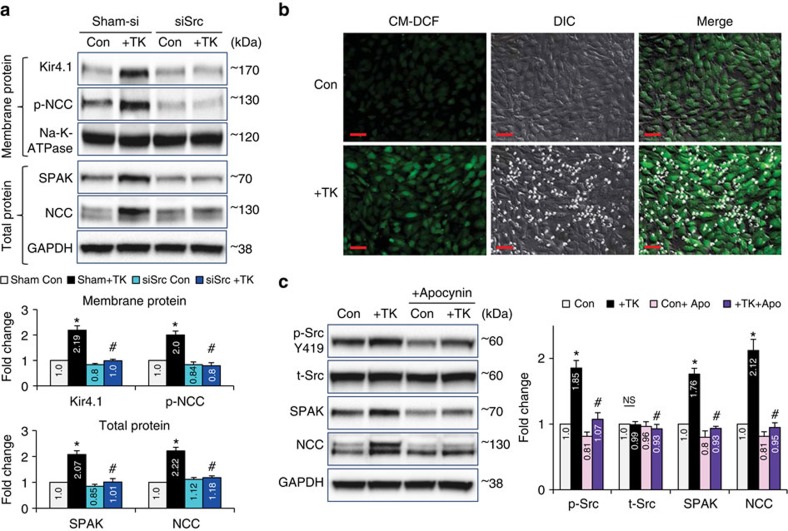
Effects of Src knockdown or blocking ROS on TK-induced NCC up-regulation in mDCTs. (**a**) Knockdown of Src using siRNA prevented TK-induced up-regulation of Kir4.1 & p-NCC on cell membrane (upper) and SPAK and NCC in total lysate (lower) of mDCT cells. Na-K-ATPase or GAPDH were used as loading controls for membrane protein or total protein, respectively. *n*=4 in each group. Data are means±s.e. *P<0.01 versus Control; ^#^*P*<0.01 versus sham+TK (ANOVA). (**b**) intracellular ROS indicator CM-DCF pre-loaded mDCTs with or without TK co-culture. CM-DCF, green; DIC, bright field; Merge, green+bright field. Images were taken using Axio observer microscopy. Scale 40 μm. Data are representative of 9 images in control group and 15 images in +TK group. (**c**) NADPH oxidase inhibitor Apocynin (100 μM) blocked TK-induced activation of Src (p-srcY419) and up-regulation of SPAK and NCC in mDCTs. Total Src (t-Src) was unaffected. GAPDH was used as loading control. *n*=4–5 in each group. Data are means±s.e. *P<0.01 versus Control; ^#^*P*<0.01 versus +TK (ANOVA).

Src kinase (Src) has been reported as an up-stream regulator of Kir4.1 in DCTs (ref. 29). It is well known that Src is activated by intracellular reactive oxygen species (ROS)^50^,^51^. Therefore, we explored whether ROS-induced Src activation is involved in CD8^+^T-induced enhancement of the Kir4.1-NCC pathway in mDCTs. To test this hypothesis, Src siRNA was used to knockdown Src in mDCTs before co-culture with TKs. Knockdown of Src inhibited the TK-induced increase of Src active form p-SrcY419 in mDCTs (Supplementary Fig. 17). Importantly, knockdown of Src also prevented the TK-induced increase of Kir4.1 on the cell membrane, as well as the downstream up-regulation of NCC in mDCTs (Fig. 9a). Similar effects were observed in cells treated with the Src inhibitor PP1 (Supplementary Fig. 18), confirming the up-stream role of Src in TK-induced Kir4.1-NCC stimulation in mDCTs. Furthermore, intracellular ROS in mDCTs was evaluated by pre-loading cells with the fluorescent ROS indicator CM-DCF. Co-culture with TK cells increased ROS accumulation in mDCTs (Fig. 9b). It is noteworthy that most mDCTs with higher levels of intracellular ROS were in contact with one or more TK cells (Fig. 9b), consistent with our earlier finding that only directly contacted mDCTs exhibited up-regulated NCC and sodium retention. To confirm the role of intracellular ROS, co-cultured cells were treated with the cell-permeable NADPH oxidase inhibitor apocynin. Apocynin diminished TK-induced Src activation and downstream up-regulation of SPAK-NCC in mDCTs (Fig. 9c). These data suggest that during CD8^+^T-DCT interaction the DCTs are signalled by ROS-induced Src activation to stimulate the downstream Kir4.1-NCC pathway.

**Figure 10:**
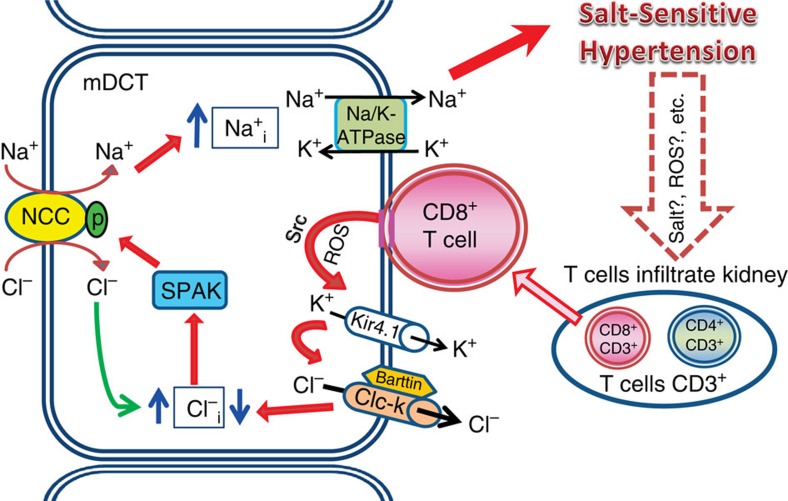
A hypothetical mechanism in kidney for the pathogenesis of salt-sensitive hypertension. Kidney infiltrated CD8^+^ T cells directly contact DCT cells on the basolateral side; stimulate DCTs via ROS-Src signaling; enhance salt retention via the Kir4.1-ClC-K—SPAK—NCC activation pathway and consequently lead to the development of salt-sensitive hypertension. Red solid arrows indicate the suggested pathway in the present study; blue solid arrows show up- or down- regulation of intracellular sodium or chloride.

Taken together, we suggest that direct contact of CD8^+^ T cells with DCTs increases ROS, activates Src and stimulates Kir4.1-ClC-K pathway-mediated chloride efflux from DCTs, which decreases intracellular chloride and leads to compensatory activation of chloride influx by activating SPAK-NCC at the cost of sodium retention, consequently resulting in salt-sensitive hypertension (Fig. 10).

## Discussion

In this study, our first novel finding is that CD8^+^ T cells up-regulate and activate NCC in DCTs, which results in sodium retention and development of salt-sensitive hypertension. Recent studies have shown that T lymphocytes play an important role in hypertension, given evidence that genetic or pharmacologic inhibition of T cells lowers blood pressure in multiple animal models of experimental hypertension^33^,^35^,^36^,^37^. In this study, we confirmed a previous finding that T cells accumulate in the kidneys of salt-sensitive hypertensive animals^35^,^36^,^37^. Several factors potentially may be involved in the mechanism of T cell-infiltration in the hypertensive kidney. First, salt may directly affect immune cells, including macrophages and T cells, that participate in inflammation and immunoreactivity^48^,^52^. As the major organ of sodium handling, the kidney has high sodium content in its tissue, especially during high-salt intake, which directly may trigger accumulation of immune cells. Second, oxidative stress and its role in hypertension are well known and may contribute to recruitment of T cells. The antioxidant tempol prevents immune cell infiltration in kidney^53^, which suggests a role for ROS in recruiting T cells. Third, exciting findings regarding T cell adaptor gene SH2B3 indicate that renal immune tolerance also determines the extent of T cell infiltration in kidney^54^,^55^. Although renal T cells unquestionably contribute to hypertension, whether T cells are involved in sodium retention in the kidney has been a critical unanswered question. Given Guyton's hypothesis that hypertensive animals and individuals exhibit impaired blood pressure-induced sodium excretion because of a physiologic defect in the kidney^7^,^8^, we hypothesized that T cells in the kidney are responsible for its defect in sodium handling by maintaining high-salt retention in the distal nephron. Co-culture with CD8^+^ T cells up-regulated SPAK and increased NCC and p-NCC in mDCTs. In addition, functional studies provided evidence that CD8^+^ T cells induce high NCC-mediated sodium retention in more mDCTs. Moreover, *in vivo* study of mice undergoing CD8^+^ T cell adoptive transfer confirmed that these effects are associated with the development of salt-sensitive hypertension.

The organ-specific homing mechanism of the CD8^+^ T cells remains unclear to us. It is also uncertain whether all CD8^+^ T cells or specific subtypes of T cells within the CD8^+^ phenotype are responsible for causing these effects. A very interesting finding is that in mice receiving CD8^+^ T cells and HS diet, although HCTZ effectively lowered blood pressure via suppressing NCC, it did not affect T cell infiltration in the kidneys. Thus discontinuing HCTZ restored hypertension in these mice on a high-salt intake, implying a possibly important role for kidney resident or memory type of CD8^+^ T cells in salt-sensitive hypertension. A similar idea also was suggested by other investigators^56^; however, further explorations are needed to specify the role of memory type T cells in regulating sodium retention in the kidney. Nevertheless, CD8 represents a critical molecule, because CD8 knockout mice are protected from AngII-induced salt-volume retention and hypertension^39^, which is in accord with our observations in this study.

Our second novel finding is that CD8^+^ T cell-induced up-regulation and activation of NCC in mDCTs occur via direct cell-cell contact. We observed that CD8^+^ T cells only induce NCC up-regulation in mDCTs via direct cell-cell contact but not in co-culture where direct cell–cell contact was prevented. Very recent studies by Kamat *et al*.^40^ and Norlander *et al*.^41^ demonstrated that AngII-induced NCC up-regulation and activation in kidney are abolished in IFNγ or IL17a knockout mice. However, in the present study, neutralizing these cytokines did not inhibit CD8^+^ T cell-induced up-regulation of SPAK-NCC in mDCTs. Although cytokines may have various effects on regulating cell signalling including ROS generation, our results suggest that CD8^+^ T cell-induced increases of NCC abundance and phosphorylation in DCTs are not directly driven by soluble cytokines released from T cells but via direct cell-cell contact. Unfortunately, T cell infiltration in the kidneys of AngII-treated IFNγ-KO or IL17a-KO mice was not evaluated in the previous reports, and our findings do not rule out possible roles of these cytokines in upstream pathways, such as recruiting T cells into the kidney or activating either T cells or DCTs to initiate their interaction. Our study using super-resolution 3D-SIM suggested an immunological synapse (IS)-like direct contact between CD8^+^ T cells and DCT cells both *in vivo* and *in vitro*. Although a true IS is mainly triggered by T cell receptors and their specific antigen ligand MHCs, other molecules, such as co-stimulators, often are involved in the communication between T cell and antigen-presenting cell. Our findings do not definitively support the formation of a true IS between these two cell types, since we have not identified the responsible co-stimulators or determined that adhesion onto mDCTs leads to activation of TKs. Whether there are other molecules involved in the crosstalk between CD8^+^ T cells and mDCTs and how this IS-like direct contact induces ROS accumulation in DCTs are two important questions for future investigation.

Our third novel finding is that CD8^+^ T cell-induced up-regulation of NCC is mediated by ROS-Src activation, thereby increasing chloride efflux via the regulation of potassium channel Kir4.1 and subsequently the chloride channel ClC-K on the basolateral cell membrane. It has been reported that intracellular chloride concentrations modulate the regulatory pathway of NCC and NKCC (refs 17, 18, 19). We detected higher membrane expression of ClC-K and binding with its beta sub-unit barttin in TK-treated mDCTs, suggestive of enhanced efflux of intracellular chloride. Supportively, blocking NCC/NKCC-mediated chloride influx unmasked the chloride efflux mediated by ClC-K, resulting in a sharp decrease in intracellular chloride in TK-treated mDCTs compared with control cells. Considering our findings in light of previous reports, we speculate that NKCC also is up-regulated by CD8^+^ T cells. Although we only detected an insignificant increasing trend of NKCC abundance in mDCTs, this could be because NKCC is mainly expressed in an earlier nephron segment than the DCT, or T cells stimulate NKCC activity rather than expression. Cells from the thick ascending limb need to be assessed to further address this question.

Using siRNAs against both ClC-K1 and 2 gave a better knockdown effect compared with either single siRNA, suggesting that either both isoforms are expressed in mDCTs or a compensatory effect exists between the two isoforms. Knockdown of both ClC-Ks largely restored the intracellular chloride concentration in TK-treated mDCTs and also abolished TK-induced NCC up-regulation and activation. These results suggest ClC-K plays a major role in TK-mediated chloride efflux and NCC up-regulation. One might speculate that sodium influx and chloride efflux must somehow be accompanied by potassium efflux to preserve charge neutrality, prevent cell depolarization and provide the driving force for chloride exit via ClC-K. We detected an up-regulation of basolateral potassium channel Kir4.1 on plasma membranes of TK-treated mDCTs, knockdown of which prevented the effects of TK-induced stimulation on ClC-K and NCC in DCTs. These results are consistent with the findings by Zhang *et al*.^30^ using Kir4.1-knockout mice, however, in a reverse direction. Our observations further strengthen the critical role suggested by these authors for Kir4.1 in regulating chloride and sodium handling in DCTs. Our observation is novel in its own right, and it is relevant for far more individuals with salt-sensitive hypertension than its earlier implication in salt-wasting syndrome.

In addition, extracellular potassium has been reported as an important factor that modulates sodium balance via regulating NCC in DCTs (refs 27, 57, 58). Hypokalemia is often seen in salt-sensitive hypertensive animals with excess mineralocorticoid or on a high-salt/low potassium diet^27^,^59^,^60^. On the other hand, hypokalemia also is a common side-effect of treatment with the NCC-inhibitor thiazide, which may be an indirect effect of delivering sodium and volume to later segments in the nephron^61^. In our study with mice receiving adoptive transfer of CD8^+^ T cells, we did not observe hypokalemia ether *in vitro* or *in vivo*. However, the pathogenesis of salt-sensitive hypertension is heterogeneous. We cannot exclude the possibility that in the progression of salt-sensitive hypertension, both CD8^+^ T cell-stimulation and hypokalemia may have a combined effect contributing to the up-regulation of NCC and further elevated blood pressure, because of high-salt intake *per se* having the effect of lowering plasma potassium^62^.

Collectively, our data suggest a novel role for CD8^+^ T cells in maintaining sodium retention in the kidney during the development of salt-sensitive hypertension, which may contribute to the kidney defect responsible for impairing blood pressure-induced sodium excretion. Moreover, we have determined the mechanism for this effect: direct contact by CD8^+^ T cells increases ROS, activates Src, stimulates Kir4.1 and upregulates ClC-K in the mDCT cell membrane, thereby increasing chloride efflux. This event leads to compensatory chloride influx via SPAK-mediated NCC-activation at the cost of increasing sodium influx and sodium retention (Fig. 10). Hence, we suggest that blocking this pathway may represent a potential new therapeutic strategy for the treatment of salt-sensitive hypertension. Moreover, our study suggests that adaptive immunity is possibly involved in the kidney defect which impairs renal electrolyte haemostasis, thereby providing novel insight into the pathogenesis of salt-sensitive hypertension.

## Methods

### Animals

The animal use protocol was approved by the Institutional Animal Care and Use Committee at University of Arkansas for Medical Sciences. In the DOCA-salt mouse model, 10 week old male C57B6 mice were uninephrectomized and randomly assigned to sham group or DOCA-salt group that received a DOCA pellet (50 mg, M-121, IRA) subcutaneously together with 1% NaCl in the drinking water for 3 weeks. In the Dahl rat model, 8 week old male Dahl salt-sensitive/resistant rats were fed a normal diet (0.3% NaCl) or high-salt diet (8% NaCl) for 4 weeks. Blood pressure in both animal models was measured directly by blood pressure catheter. In the CD8^+^ T cell adoptive transfer mouse model, uninephrectomized 12 weeks old male C57B6 mice were randomly assigned to sham group with saline injection or adoptive transfer of CD8^+^ T cell group that was injected with freshly isolated CD8^+^ T cells (from spleens of DOCA-salt mice) via one shot tail vein injection at the dose of 3 × 10^7^ cells/100 μl saline per mouse. High-salt diet (8% NaCl) or HCTZ drinking water (0.3 g l^−1^) were given to sham and mice receiving CD8^+^ T cells as indicated in Fig. 3c (also in Supplementary Fig. 5). Blood pressure in this mouse model was measured directly by radio-biotelemetry (DSI).

### Splenic T cell isolation

Mouse spleens were dissociated using a GentleMACs tissue dissociator. Dynabeads T cell isolation kits (pan, CD4^+^, CD8^+^) were used to isolate T cells from splenocytes. All procedures followed manufacturer's protocol. Subtypes of collected T cells were confirmed using flow cytometry.

### Cell tracker labelling CD8^+^ T cells

Freshly isolated mouse CD8^+^ T cells were incubated with CellTracker Red CMTPX dye (Molecular Probe) at 25 μM for 40 min in 37 °C incubator with gentle rotating. After labelling, cells were washed with PBS and injected into mice immediately.

### Cell culture treatment & harvest

All cells were maintained at 37 °C and 5% CO_2_. The mDCT15 cell line (they will be referred to in the text simply as mDCTs), a recently established active and stable model of mouse distal convoluted tubule^44^, was cultured in DMEM/F12 media with 5% FBS. Passages 11–25 were used in this study. The TK cells were purchased from ATCC (CRL-2396) and maintained in RPMI1640 medium with 10% FBS. Passages 4–16 were used in this study. No contaminations were found in culture cells throughout all experiments. For co-culture study, mouse splenic T cells (pan, CD4^+^, CD8^+^) were directly added to mDCTs at a concentration of 3 × 10^6^/ml; TKs were administrated to mDCTs at 2 × 10^6^ ml^−1^ directly or indirectly (using transwell inserts with 0.4 or 8 μm pore size). After overnight co-culture, cells were washed with PBS (most suspension T cells can be removed at this step). Then, mDCTs from all groups were scraped in cell isolation buffer (EDTA 2 mM+0.25%BSA in PBS) and were subjected to specific magnetic dynabeads to deplete remaining T cells contaminating the mDCTs. Pure mDCTs were confirmed by flow cytometry with no CD3 staining and thereafter used for function study, extraction of total protein in RIPA buffer; isolation of membrane protein using Mem-PER Membrane Protein Extraction Kit (Pierce) following manufacturer's protocol or extraction of mRNAs using RNeasy plus kit (QIAGEN).

### Potassium measurement

Potassium concentration in mouse plasma and cell culture media was measured using i-STAT blood analyser with EC8+ cartridges following the manufacturer's protocol.

### Super-resolution 3D structured illumination microscopy

Raw images were acquired on a Zeiss Axio Observer Z1 SR using ELYRA PS.1 structured illumination microscopy with a × 100/1.46 Plan Apochromatic objective and a PCO scientific CMOS camera. Four solid-state lasers (405, 488, 561 and 647 nm) were used to take 40–60 Z-slices at 116 nm interval and five grid rotations. Images were acquired and processed with Zeiss Zen Black 2.1 software. Channel alignment for chromatic aberration was performed using multispectral beads.

### Flow cytometry

For surface staining, cells were stained with antibodies (CD3, CD4, CD8, MHC-I, MHC-II from BD Biosciences or Biolegend, dilution 1:200) for 30–45 min in cell isolation buffer in dark tubes before analysis. For NCC function study using CoroNa Green (cell permeable, Molecular Probes), mDCTs were treated with ouabain, bumetanide and amiloride for 30 min at room temperature (RT) followed by loading CoroNa Green at a concentration of 10 μM for 1 h at RT. Cells were washed and re-suspended in PBS containing the same blockers mentioned above for 45 min and analysed in a BD Accuri C6 flow cytometer immediately. Flow cytometry data were analysed using FlowJo software.

### Measurement of NCC-mediated sodium uptake using CoroNa Green

The same procedures were used as in the CoroNa Green flow cytometry study, except that ±HCTZ (200 μM) was added together with the other sodium transporter blockers and ±40 mM additional NaCl was added to PBS after CoroNa Green loading. After incubation, cells were lysed by sonication in buffer containing 50 mM MOPS, 100 mM KCl and 1% NP40. Fluorescence units were measured using a Glomax multi meter and normalized by the protein concentration of each sample.

### Intracellular chloride measurement using MQAE

Cell permeable MQAE was purchased from Molecular Probes. Harvested mDCTs were incubated in Cl^−^ free PBS (Cl^−^ was replaced by NO_3_^−^) with or without HCTZ/bumetanide for 30 min followed by loading MQAE at a 5 mM concentration for 1 h. Cells were washed and re-suspended in Cl^−^ free PBS (F0) or PBS (Ft) (with or without HCTZ/bumetanide) for 45 min, then lysed by sonication in Cl^−^ free PBS+1% NP-40. Fluorescence units were measured immediately using a Glomax multi meter and normalized by protein concentration of each sample. Ratio of intracellular chloride concentration was calculated by equation (F0/Ft)−1 as in the manufacturer's protocol.

### Individual cell staining

mDCTs were scraped into culture media and mixed with TKs. The cell mixture was incubated with rotating in 37 °C incubator for 4 h then seeded in to poly-D-lysine pre-coated imaging dish, followed by washing, fixing and staining with fluorescent labelled antibodies. Images were taken using super-resolution 3D-SIM microscopy.

### Intracellular ROS detection using CM-DCF

mDCT cells were pre-loaded with CM-DCF (Molecular Probe) at 10 μM in 37 °C incubator for 35 min. After washing, cells were treated with TKs for 5 h and followed by washing with PBS. During the time of imaging, cells were maintained in Live Cell Imaging Solution (Molecular Probes).

### Transfection of siRNAs

All siRNAs were purchased from ABI. Transfection of siRNAs was performed using lipofectamine RNAimax following the manufacturer's protocol.

### Western blot

4–12%, 4–20% or 8% Bis–tris gel (all from Genscript) were selected according to the molecular weight of the target protein. After electrophoresis, gels were transferred to PVDF membranes on ice. All membranes were blocked in 5% non-fatty milk. Information for primary and secondary antibodies is provided in Supplementary Table 1. Western blot of NCC detected two bands in the range of 100–140 KDa, indicating mature (glycosylated) and immature (un-glycosylated) forms of NCC (refs 63, 64). Western blot images were obtained using a ChemiDoc XRS+ system and analysed using ImageLab software (BIORAD). Full images of all blots are provided in supplementary Fig. 19. Fold changes are relative to control/loading (defined as 1.0).

### Immunostaining

Following antigen retrieval using antigen unmasking solution (Vector), sections were washed and blocked in 5% non-fatty milk containing 4% goat serum at RT for 1 h. Antibodies are described in Supplementary Table 1. After staining, sections were sealed with ProLong Gold mounting media (Molecular Probes). Fluorescent immunostaining images were captured using an Axio Imager microscope (Zeiss).

### Realtime PCR

RNA reverse-transcription was performed using Oligo dT and Superscript III system (Invitrogen) following the manufacturer's protocol (except incubation time was extended to 90 min). All Taqman realtime PCR primers were purchased from ABI. Realtime PCR tests were performed using an Mx3005P system (Agilent Tech) and data were analysed using the ΔΔCt method.

### Membrane protein immunoprecipitation (IP)

In this study, IP was used to detect binding between ClC-K and barttin on the mDCT cell membrane. Membrane protein from each group was adjusted to 750 μg in 500 μl solution. 30 and 5 μg protein from each group was utilized for western blot for ClC-K and Na-K-ATPase, respectively; the remaining protein was incubated with mouse monoclonal barttin antibody (Santa Cruz, 1:50) or rabbit polyclonal ClC-K antibody (Alomone, 1:50). After incubation, barttin-ClC-K complex was pulled down using IgG magnetic beads and detected by immunoblot.

### Statistical analysis

Data are presented as means±s.e. Unpaired Student's *t*-test was used for comparisons between two groups. For multiple comparisons, statistical analysis was carried out by ANOVA followed by Tukey's or Dunnett's post hoc tests. *P* values<0.05 were considered to be significant. No sample size estimate was performed, but sample size was selected based on previous experiments. All assays were repeatable in independent experiments and displayed figures are representative.

### Data availability

All relevant data are available from the authors on request.

## Additional information

**How to cite this article:** Liu, Y. *et al*. CD8^+^ T cells stimulate Na-Cl co-transporter NCC in distal convoluted tubules leading to salt-sensitive hypertension. *Nat. Commun.* **8,** 14037 doi: 10.1038/ncomms14037 (2017).

**Publisher's note**: Springer Nature remains neutral with regard to jurisdictional claims in published maps and institutional affiliations.

## Supplementary Material

Supplementary Information Supplementary Figures and Supplementary Table.
