# Supplementary material for: CD8+ T cells stimulate Na-Cl co-transporter NCC in distal convoluted tubules leading to salt-sensitive hypertension
Source: Nat Commun. 2017 Jan 9;8:14037. doi: 10.1038/ncomms14037 (PMC5227995; doi:10.1038/ncomms14037)
Supplement: Supplementary Information — Supplementary Figures and Supplementary Table. [file ncomms14037-s1.pdf]

Supplementary Figure 1 (Mu)

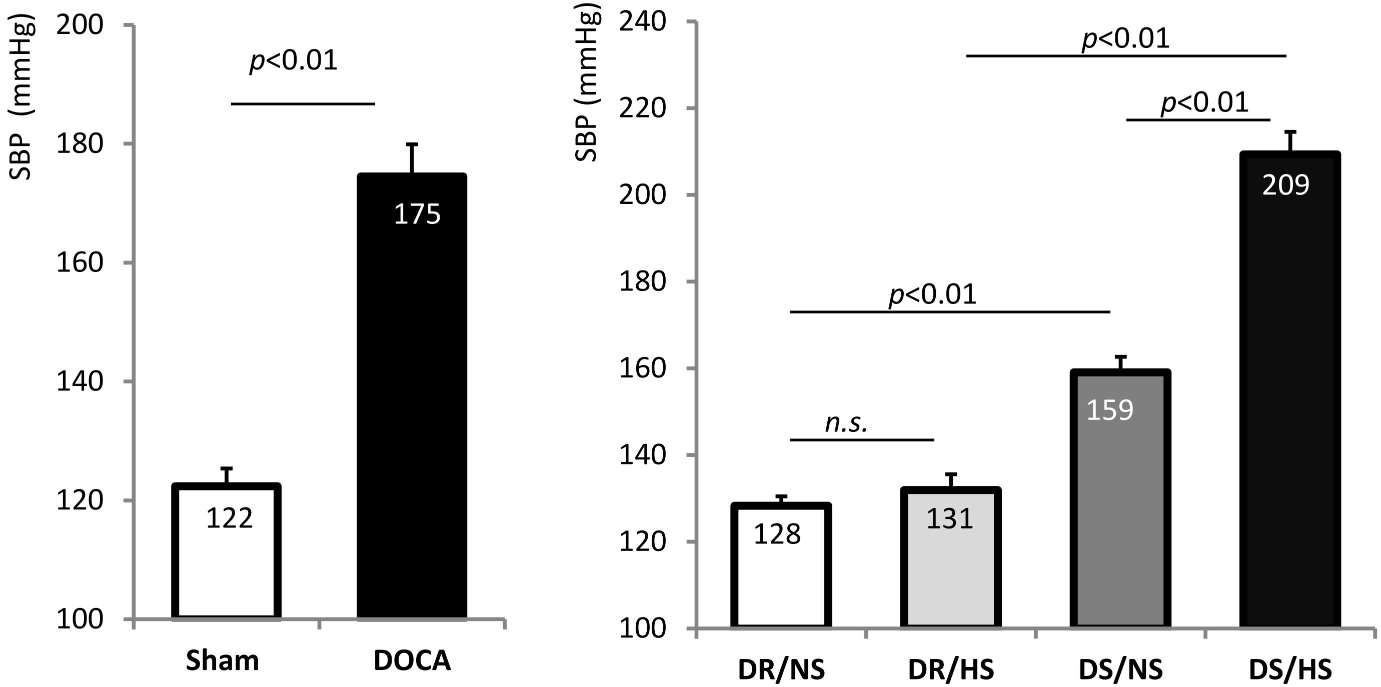

Supplementary Figure 1 Systolic blood pressure (BP) in DOCA mice or Dahl rats was measured directly using telemetry/catheter via the left carotid artery at the end of the treatment (DOCA mice 3 weeks; Dahl rats 4 weeks). n=4-6 animals for each group. Data are means ± s.e. $p<0.01$ (t-test for DOCA groups; ANOVA for Dahl groups).

Supplementary Figure 2 (Mu)

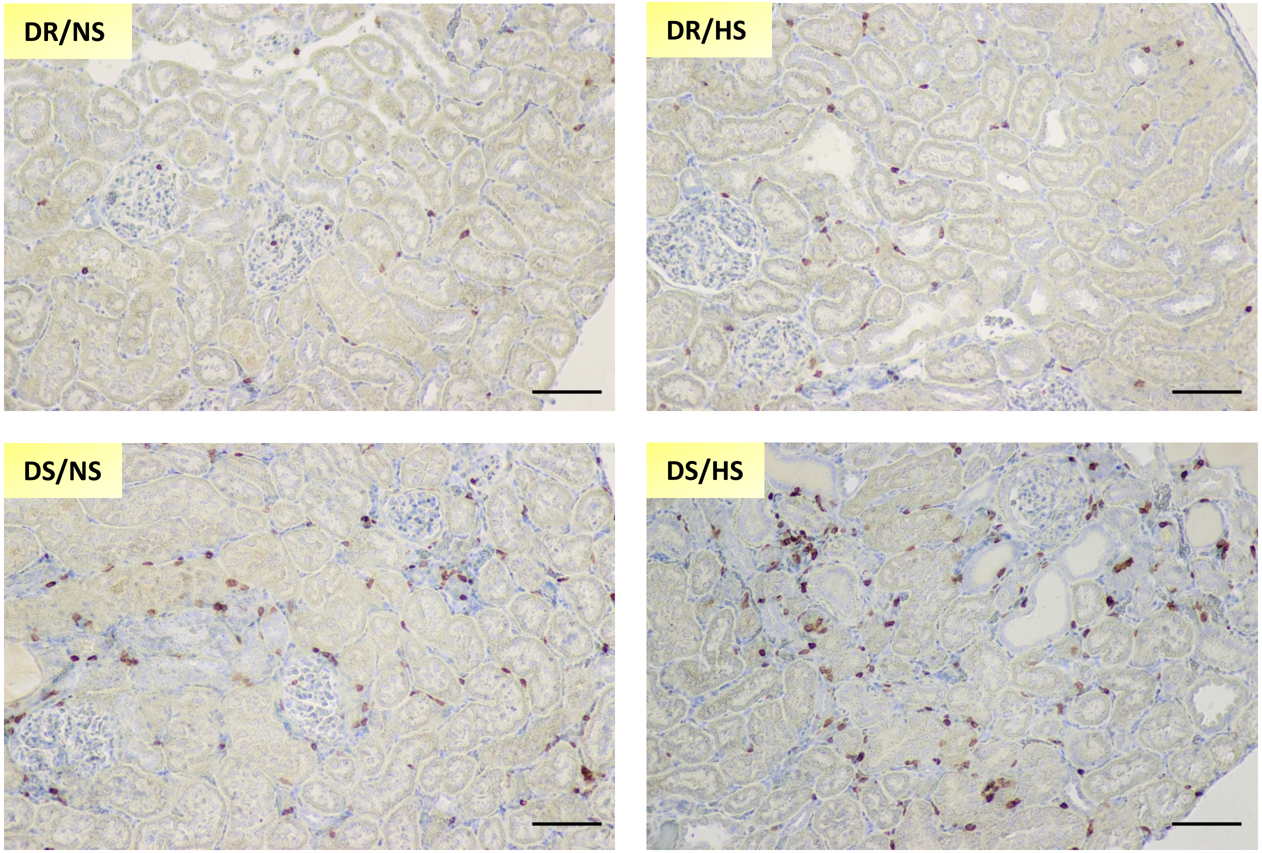

Supplementary Figure 2 Immunostaining of pan T cell marker CD3 (brown) on kidney sections of Dahl salt resistant (DR) / sensitive (DS) rats with high salt (HS) or normal salt (NS) diet for 4 weeks. Nuclei were stained by Hematoxyline (blue). Data are representative of n=8 images in each group. scale 50µm.

Supplementary Figure 3 (Mu)

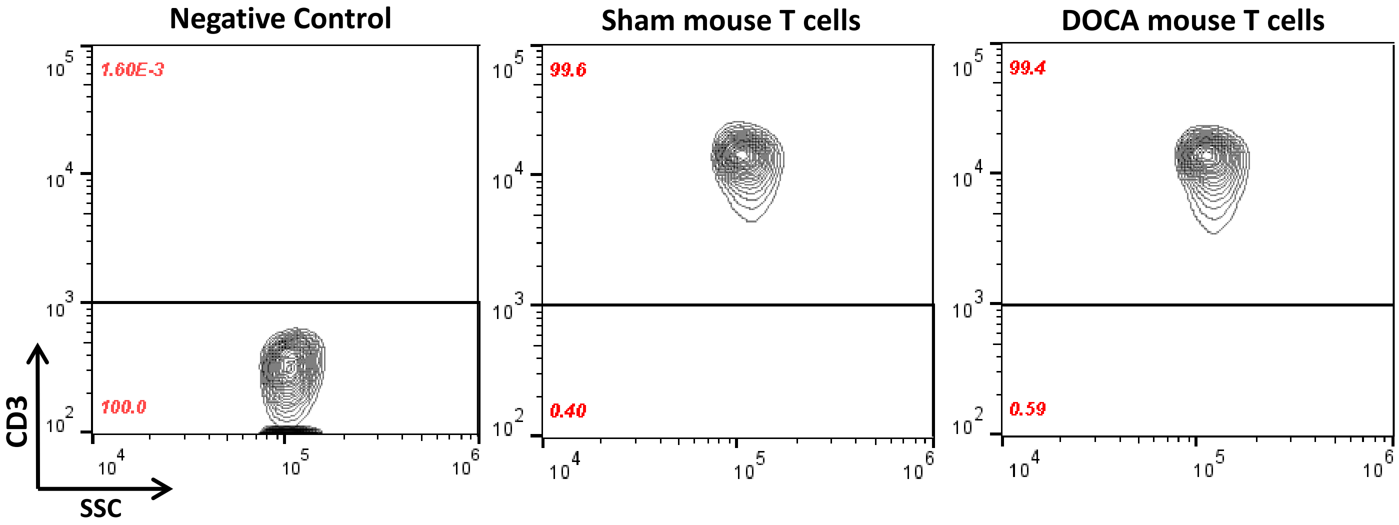

Supplementary Figure 3 Splenic pan T cells isolated from Sham mice or DOCA mice were stained with CD3 antibody. A mixture of cells without CD3 staining from both groups of mice was used as negative control. Flow cytometry confirmed all cells isolated from spleen from either set of mice are $CD3^+$ T cells. Red numbers indicate the proportion (%) of $CD3^+$ (upper) and $CD3^-$ (lower) cells in each group. Data are representative of three independent experiments.

Supplementary Figure 4 (Mu)

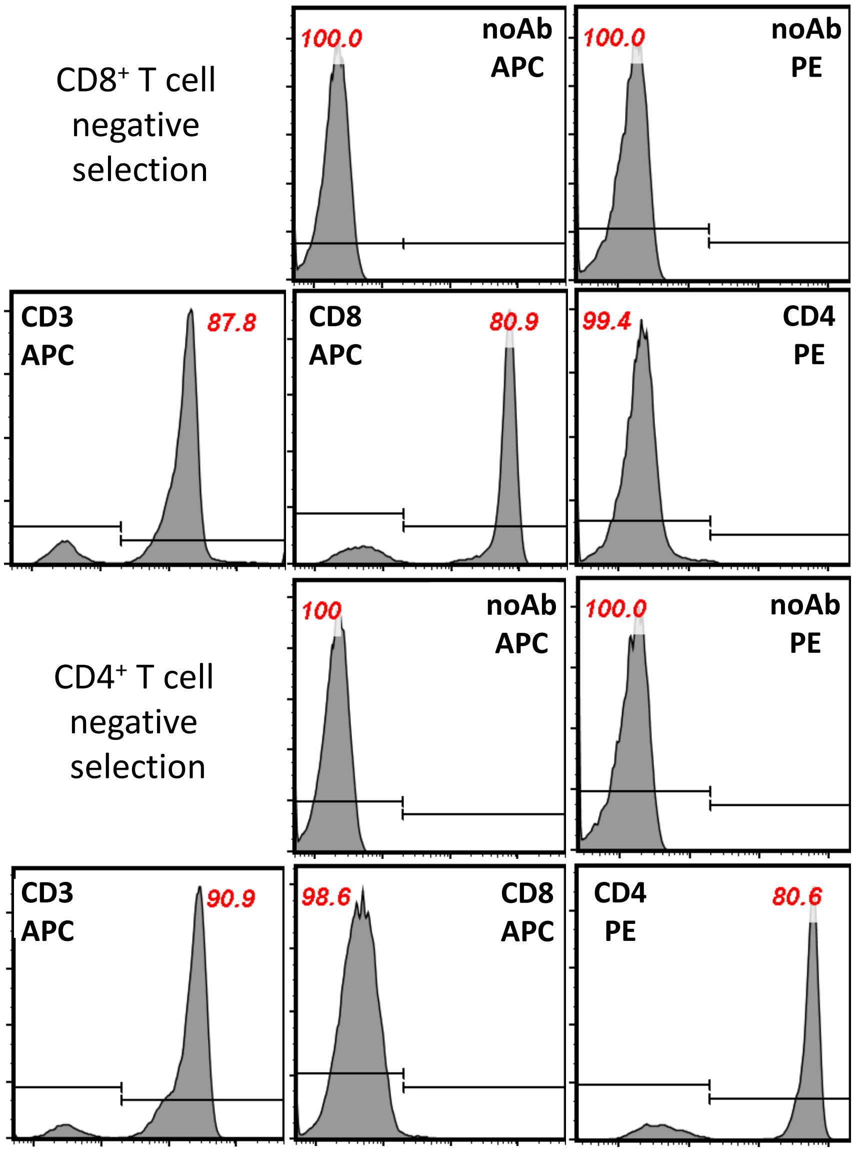

Supplementary Figure 4 Mouse CD8⁺ (upper panels) and CD4⁺ (lower panels) positive T cells isolated from mouse spleens using negative selection were stained with CD3, CD4 and CD8 antibodies. Flow cytometry confirmed the purity of both isolated cells was higher than 80%. Neither contamination of CD4⁺ T cells in isolated CD8⁺ T cells, nor CD8⁺ T cells in isolated CD4⁺ T cells, were detected. Red numbers indicate the proportion (%) of cells in indicated area. Data are representative of three to four independent experiments.

Supplementary Figure 5 (Mu)

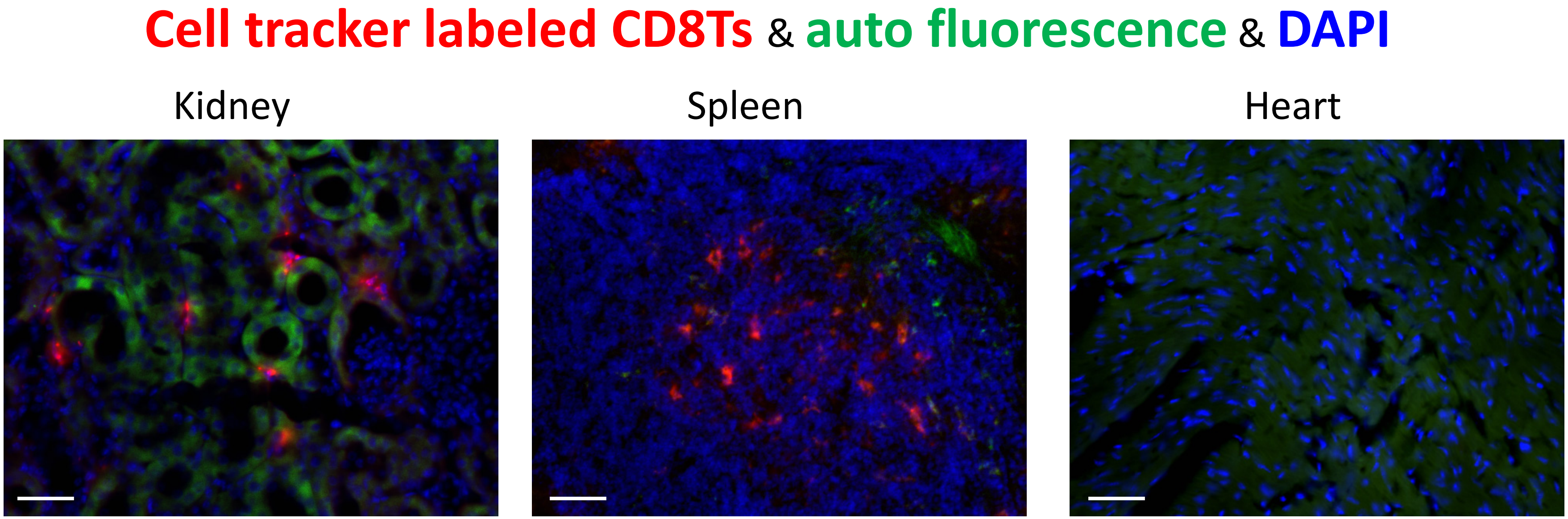

Supplementary Figure 5 Fresh isolated CD8$^+$ T cells were pre-loaded with fluorescent Cell Tracker (red) before adoptive transfer to mice. Due to the limit of fluorescent dye bleaching, mice receiving cells were sacrificed in 72 hours (fed on regular diet for 40 hours followed by HS diet for 32 hours) after the adoptive transfer of fluorescent labeled CD8$^+$ T cells. The kidneys, spleens and heart were fixed, embedded in OCT and sliced on a cryostat. Exogenous CD8$^+$ T cells were found in the kidneys, the spleens but not the hearts in the adoptive transfer-receiving mice. Red color is fluorescent cell tracker representing exogenous CD8$^+$ T cells; Green color is tissue auto-fluorescence due to PFA fixation indicating tissue morphology; Blue color is DAPI staining. Data are representative of n=9-13 images in each group. scale 50μm.

Supplementary Figure 6 (Mu)

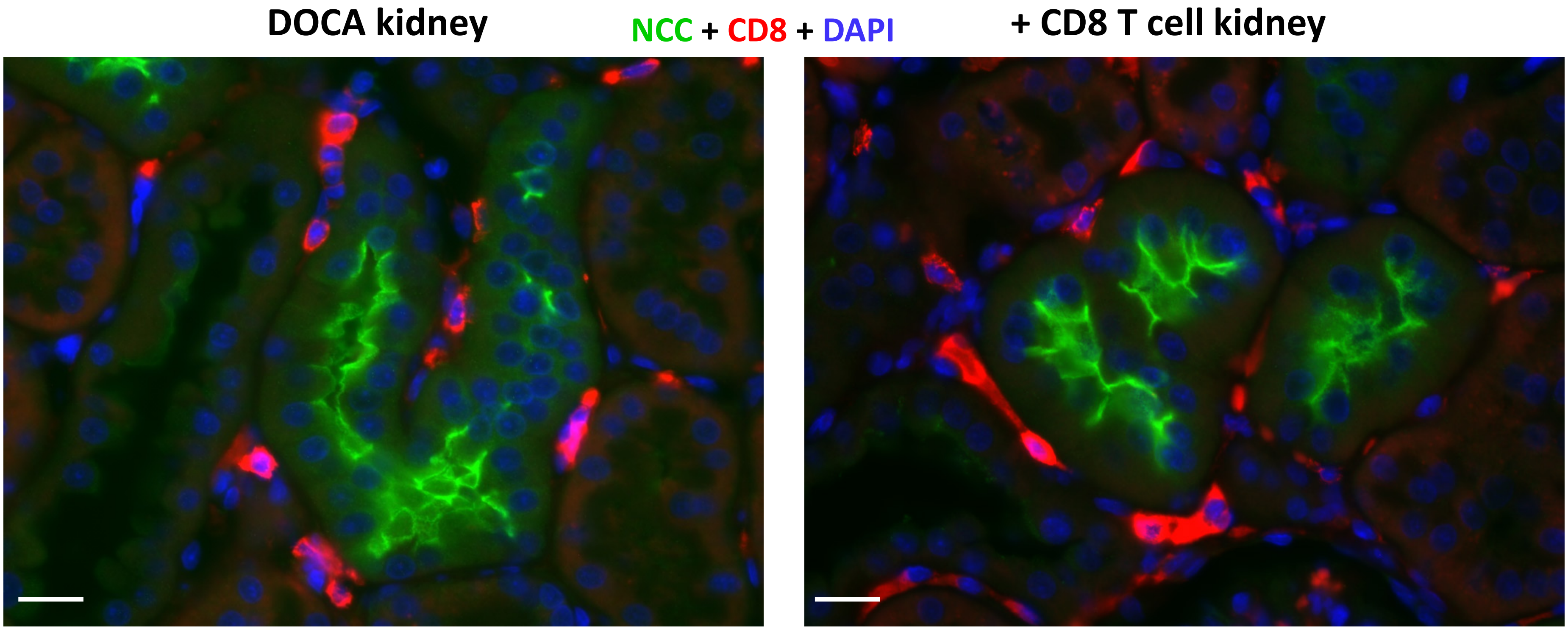

Supplementary Figure 6 Double staining of CD8 (red) and NCC (green) in the kidneys of DOCA mice (left panel) and mice receiving adoptive transfer of $CD8^+$ T cells (right panel). Nuclei were stained by DAPI (blue). Data are representative of n=12 images in each group. scale 20μm.

Supplementary Figure 7 (Mu)

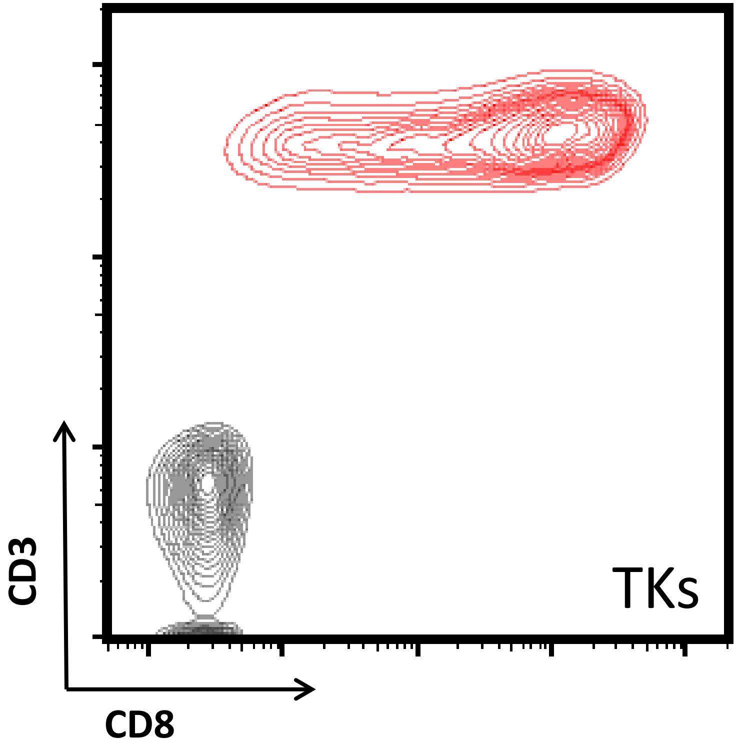

Supplementary Figure 7 Flow cytometry confirmed that TK-1 cells are $CD3^+$ and $CD8^+$. Cells without staining are labeled with gray color (negative control); Cells stained with both CD3 and CD8 antibodies are labeled with red color. Data are representative of four independent tests.

Supplementary Figure 8 (Mu)

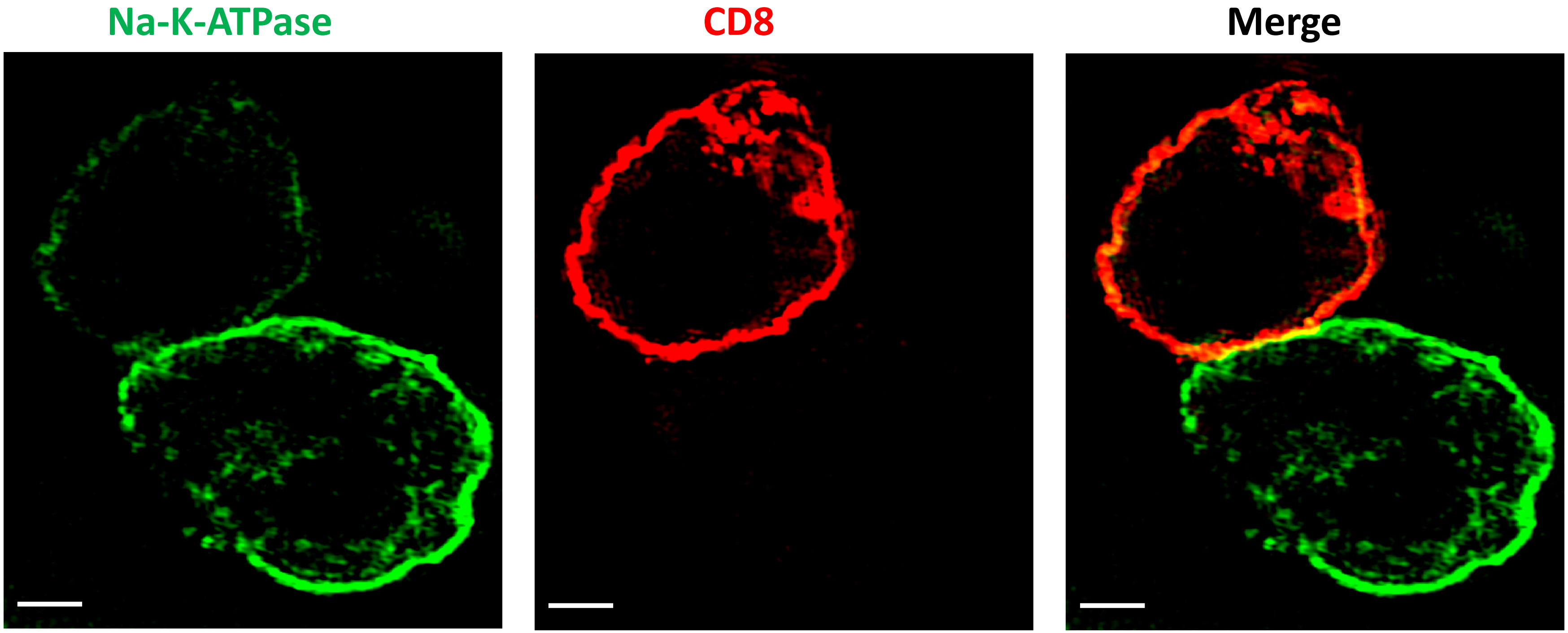

Supplementary Figure 8 Channel split images of Fig. 5b, demonstrating direct contact of TK and mDCT cells in individual cell level. As expected, the staining of Na-K-ATPase (green) on mDCT membrane was much stronger compered to TK membrane. And CD8 (red) only stained on the surface of TKs but not mDCTs. Yellow area (merge) at the cross of both cells indicates the direct contact of TK and DCT cells. Data are representative of n>15 images. Scale 2mm.

Supplementary Figure 9 (Mu)

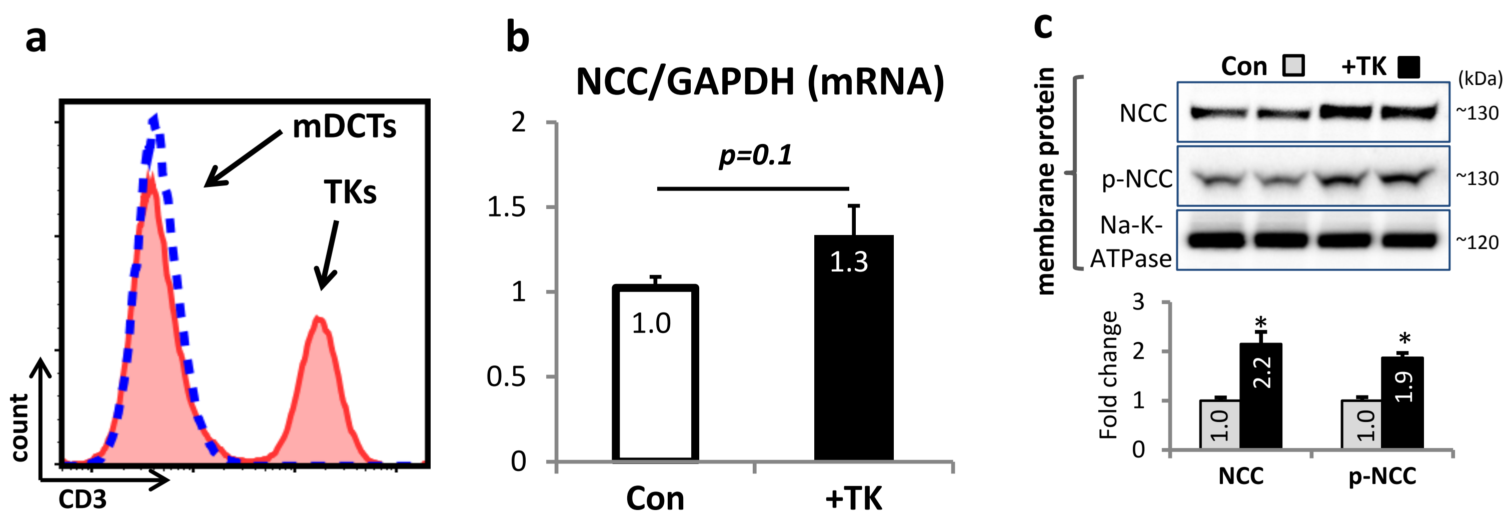

Supplementary Figure 9 Effects of co-culture on NCC expression in mDCTS. **(a)** CD8 specific magnetic dynabeads completely removed TKs from the mDCT-TK co-culture. PBS washed co-cultured cells before (red area) or after (blue dashed lined area) removal of TKs using magnetic beads were stained by CD3 antibody. Data are representative of three independent tests. **(b)** Realtime-PCR using specific TaqMan primers illustrated NCC mRNA expression level in mDCTs with or without prior TK-treatment. n=10 in each group. Data are means ± s.e. *p*=0.1 (t-test) **(c)** Membrane protein expression of NCC and p-NCC in mDCTs with or without TK co-culture. Na-K-ATPase was used as loading control. n=4-6 in each group. Data are means ± s.e. *p<0.01 vs. Control (t-test).

Supplementary Figure 10 (Mu)

a

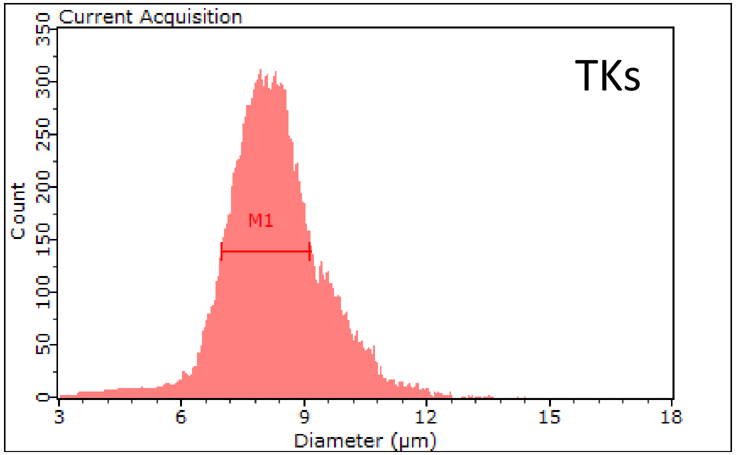

b

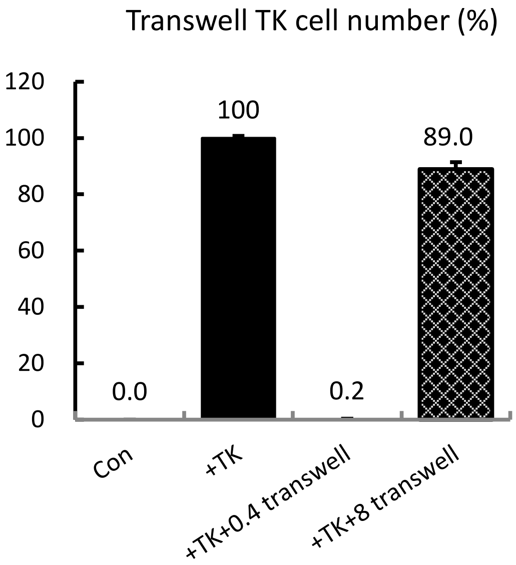

Supplementary Figure 10 Size of TKs and ytanswell ability. **(a)** TK cell size was measured by Scepter 2 cell counter. Most TK cells are about 7-9 mm in diameter. Data are representative of >10 independent tests. **(b)** Proportion of TK cells passing through transwell co-culture inserts with different pore size (0.4 mm, 8 mm). TK cell number with no transwell chamber (+TK) set as 100%. n=6 in each group. Data are means ± s.e.

Supplementary Figure 11 (Mu)

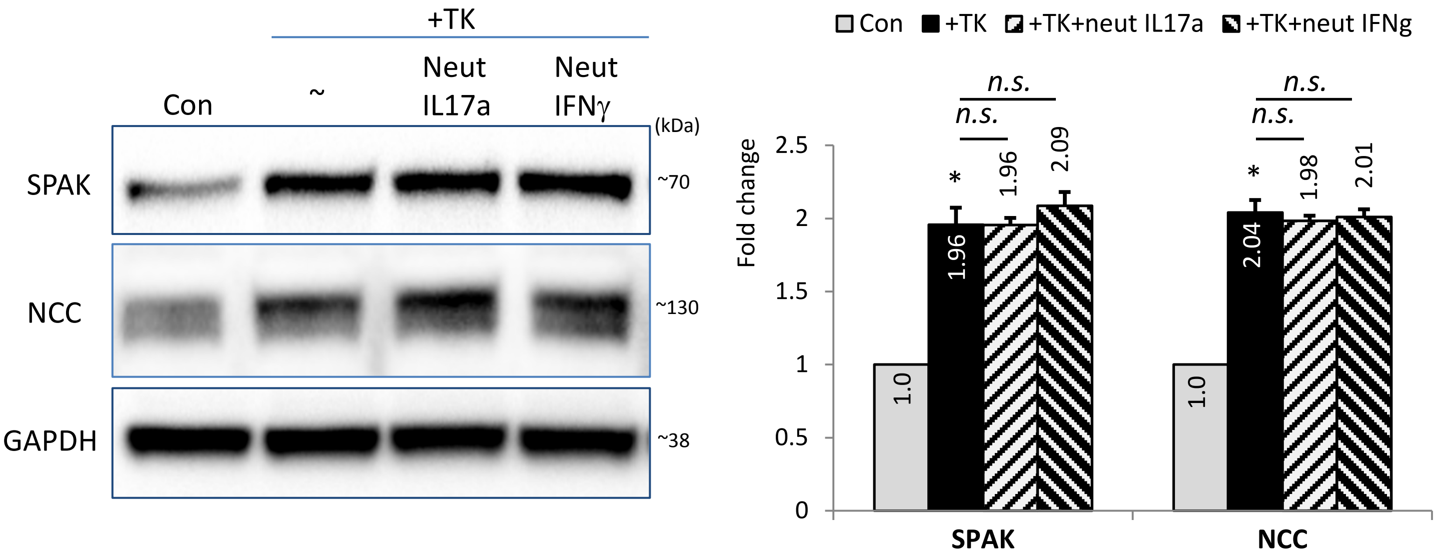

Supplementary Figure 11 Neutralizing antibodies for IL17a (10 mg/ml) and IFNg (10 mg/ml) were added to mDCT-TK co-culture (15 mins prior to adding TKs). After co-culture, mDCTs were analyzed by western blot for expression of SPAK and NCC. Neither neutralizing antibody has blocking effect on TK-induced NCC up-regulation. GAPDH was used as a loading control. n=4 in each group. Data are means ± s.e. *$p<0.01$ vs. Control (ANOVA).

Supplementary Figure 12 (Mu)

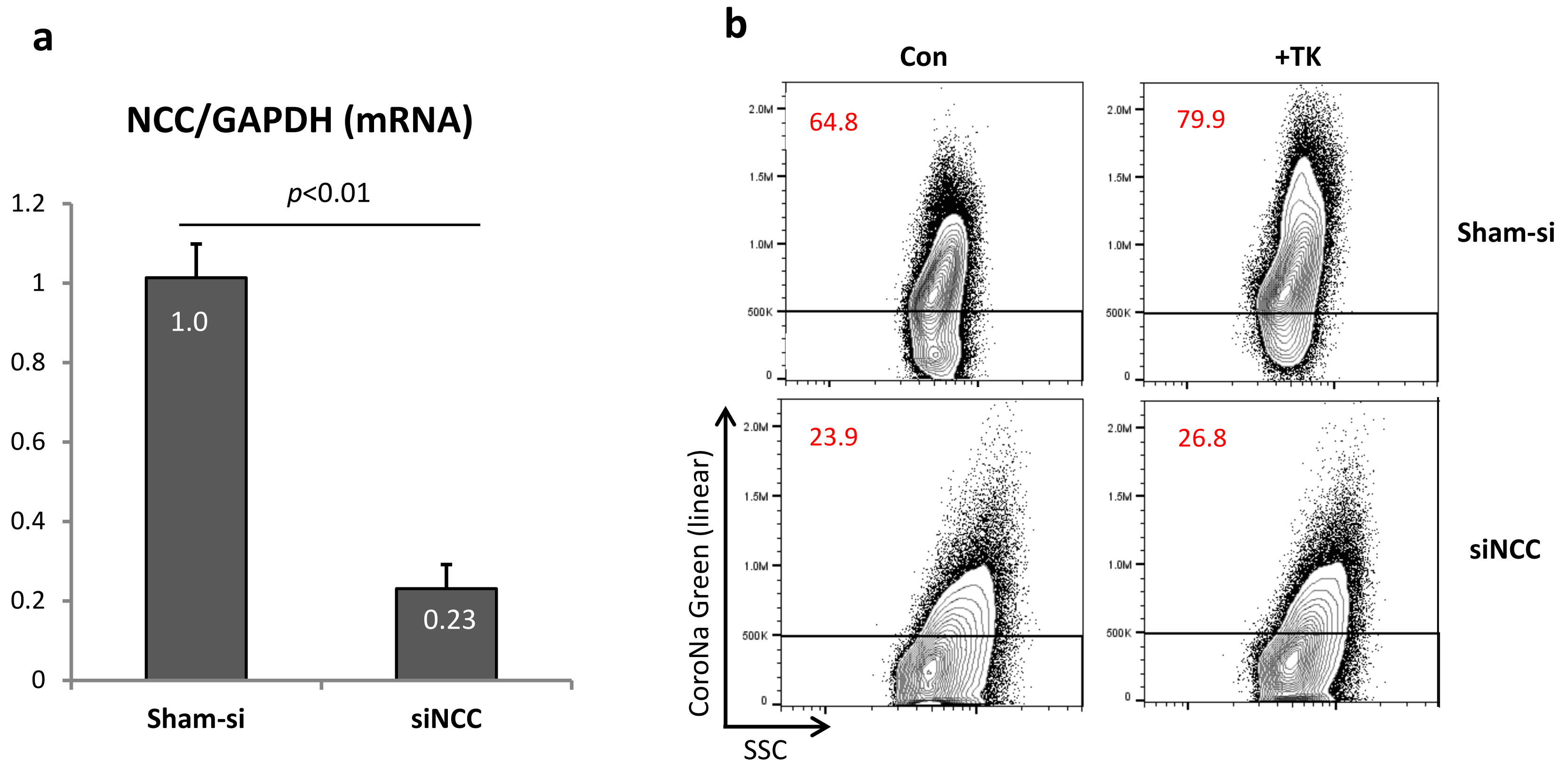

Supplementary Figure 12 Effects of NCC knockdown with siRNA. **(a)** Realtime-PCR using specific TaqMan primers detected NCC mRNA in the mDCTs with (siNCC) or without (sham-si) NCC knockdown by siRNA. n=4 in each group. Data are means ± s.e. $p<0.01$ (t-test) **(b)** NCC knockdown by siRNA (siNCC) decreased proportion of high sodium-containing mDCTs in both TK-treated and untreated groups. Moreover, siNCC abolished the effect of TK-mediated increase of sodium retention in mDCTs. Red numbers indicate the proportion (%) of cells with high sodium content. Data are representative of four independent experiments.

Supplementary Figure 13 (Mu)

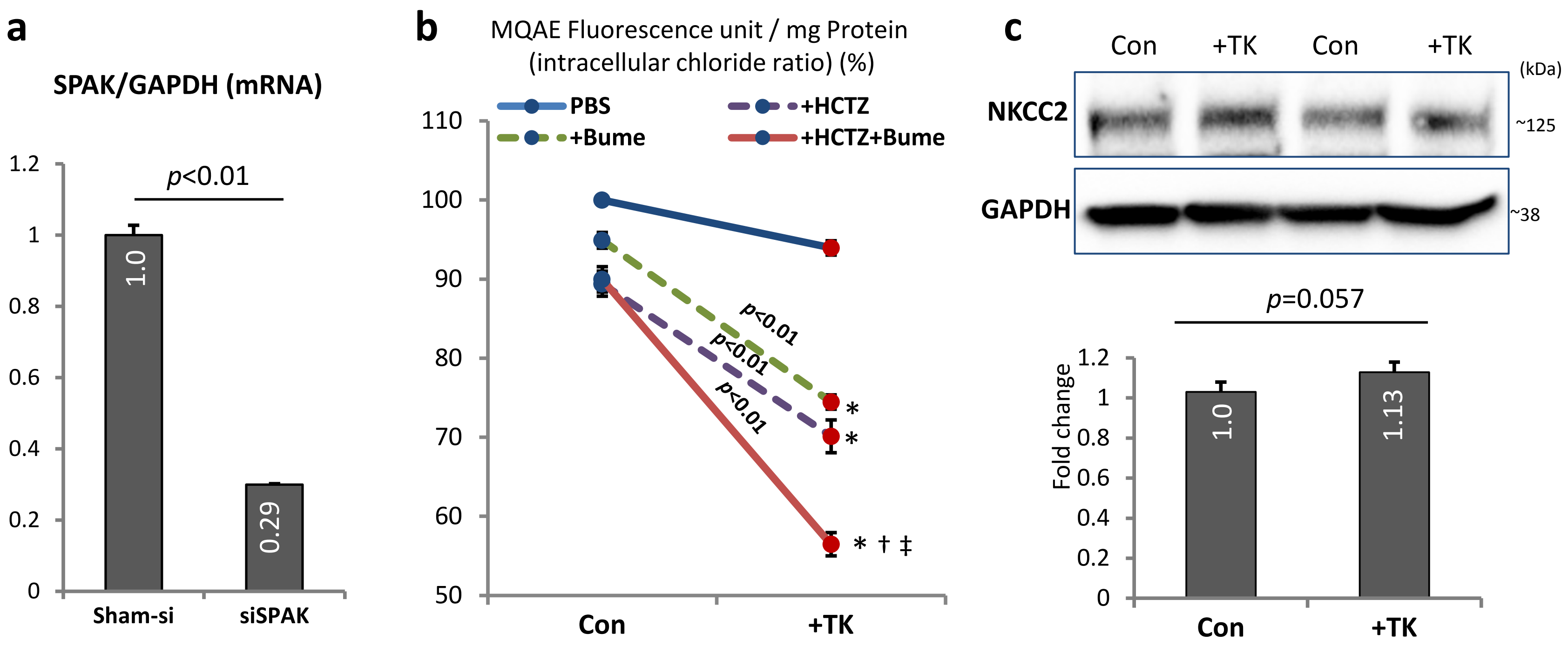

Supplementary Figure 13 Effects of co-culture on intracellular chloride and NKCC2 expression in mDCTs. **(a)** Realtime-PCR using specific TaqMan primers detected SPAK mRNA in the mDCTs with (siSPAK) or without (sham-si) SPAK knockdown by siRNA. n=4 in each group. Data are means ± s.e. $p<0.01$ (t-test) **(b)** Effect of blocking chloride influx with HCTZ (200mM) and/or Bume (100mM) on intracellular chloride concentration (ratio) in TK-treated (right, +TK) or untreated (left, Con) mDCTs. Blocking both pathways of chloride entry led to a larger decrease in intracellular chloride than blocking either pathway alone. Intracellular chloride concentration was measured with the chloride indicator MQAE and normalized per milligram protein. n=8-10 in each group. Data are means ± s.e. $p<0.01$(shown in figure, t-test); *$p<0.01$ vs. PBS+TK; †$p<0.01$ vs. PBS+TK+Bume; ‡$p<0.01$ vs. PBS+TK+HCTZ (ANOVA). **(c)** Western blot of NKCC2 (SLC12A1) abundance in mDCTs with or without TK co-culture. GAPDH was used as a loading control. n=4 in each group. Data are means ± s.e. $p=0.057$ (t-test)

Supplementary Figure-14 (Mu)

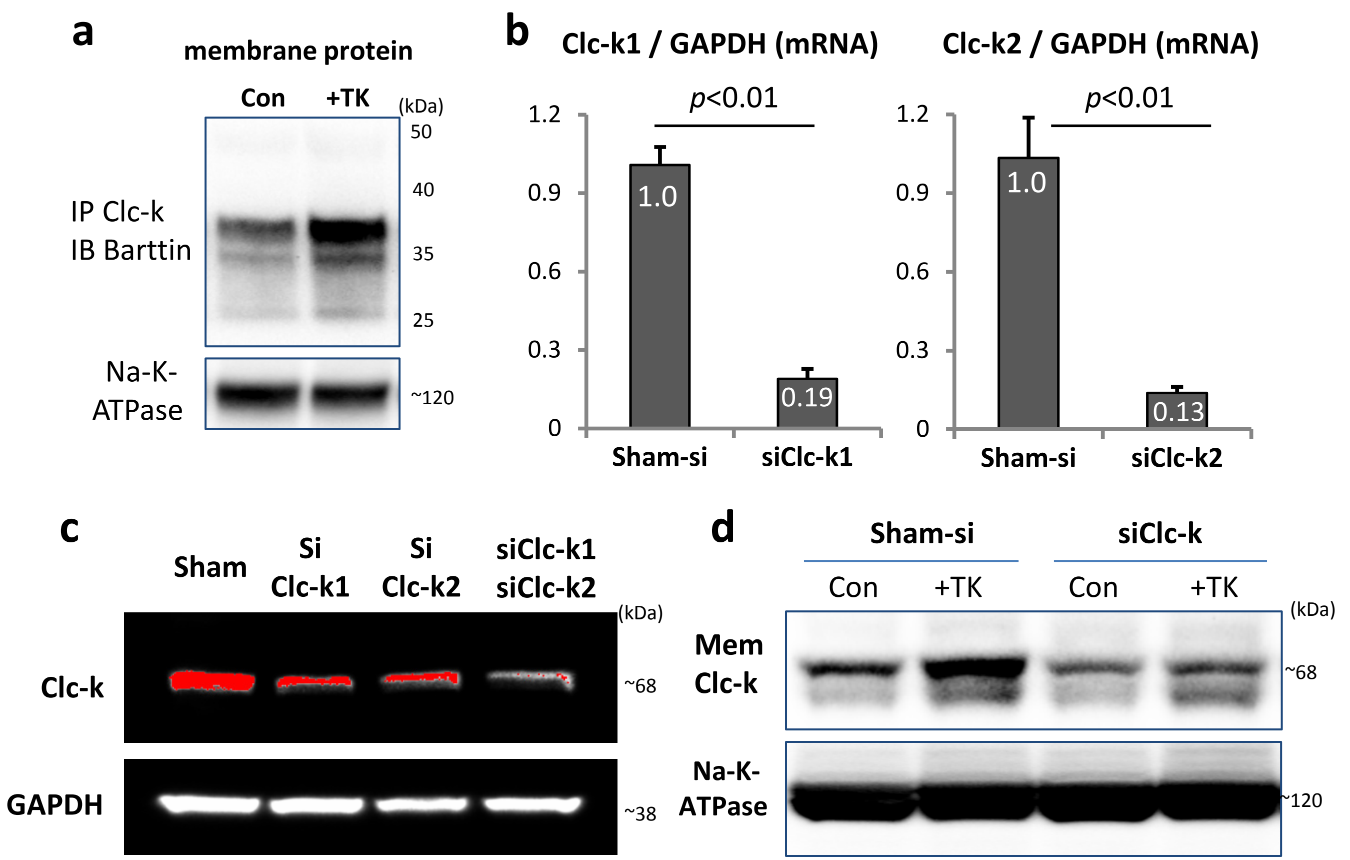

Supplementary Figure 14 Effects of Clc-K knockdown using siRNAs against both Clc-K1 and Clc-K2. **(a)** The binding of membrane Clc-k to its subunit barttin was detected by immunoprecipitation (IP) of Clc-k and immunoblot (IB) of barttin (reverse from Figure 7b). Membrane protein loading for both western blot and IP/IB were normalized by Na-K-ATPase. Data are representative of n=4 in each group. **(b)** Knockdown efficiency of siClc-ka or siClc-kb was evaluated by realtime-PCR using specific TaqMan primers (ABI) against Clc-ka or Clc-kb. n=4 in each group. Data are means ± s.e. $p<0.01$ (t-test) **(c)** In western blot, Clc-k antibody from Alomone detects both isoforms of Clc-k. Image analyzed by Image Lab software. Saturated pixels were highlighted in red. GAPDH was used as loading control. Data are representative of two independent tests. **(d)** Clc-k knockdown using both siRNAs inhibited TK-induced up-regulation of membrane expression of Clc-k. Na-K-ATPase was used as membrane protein loading control. Data are representative of n=4-6 in each group.

Supplementary Figure 15 (Mu)

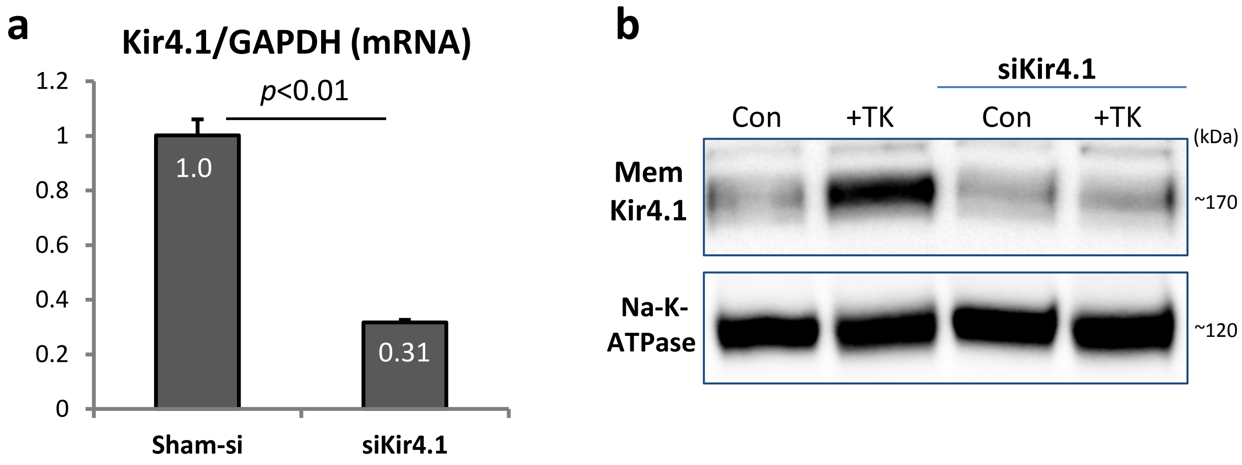

Supplementary Figure 15 Effects of Kir4.1 knockdown using siRNA. **(a)** Realtime-PCR using specific TaqMan primers detected Kir4.1 mRNA in the mDCTs with (siKir4.1) or without (sham-si) Kir4.1 knockdown by siRNA. n=4 in each group. Data are means ± s.e. p<0.01 (t-test). **(b)** Knockdown of Kir4.1 prevented TK-induced up-regulation of membrane expression of Kir4.1. Na-K-ATPase was used as membrane protein loading control. Data are representative of n=4 in each group.

Supplementary Figure 16 (Mu)

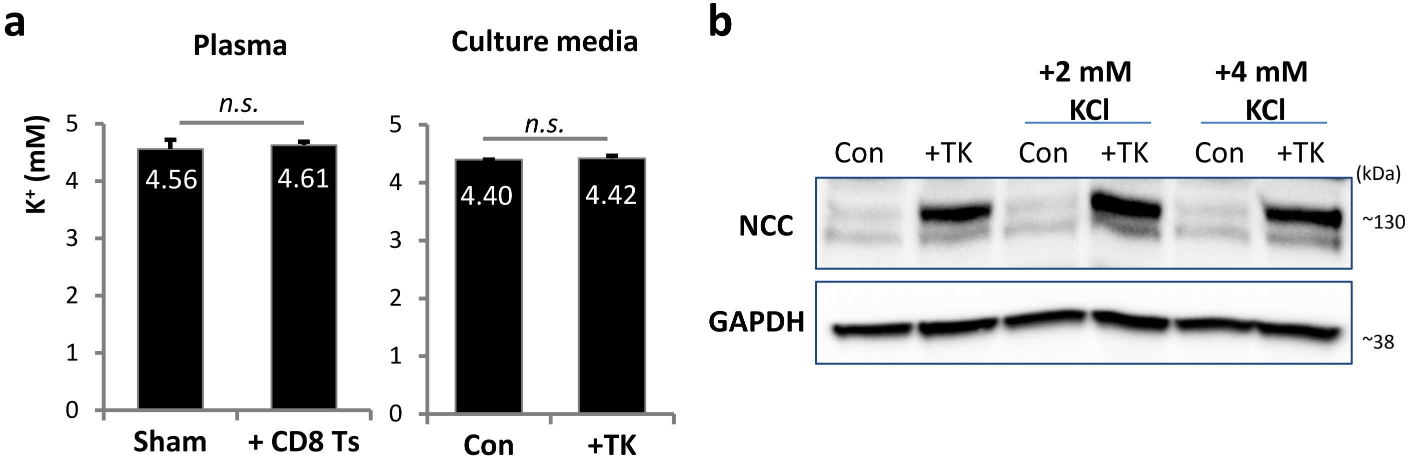

Supplementary Figure 16 Role of potassium in CD8 T cell-mDCT cell interaction **(a)** left, plasma potassium level in mice with or without receiving adoptive transfer of $CD8^+$ T cells (measured at day 3 after adoptive $CD8^+$ T cell transfer); right, potassium concentration in mDCT culture media with or without co-culture with TKs. n=4-5 in each group. Data are means ± s.e. no significance observed (t-test). **(b)** NCC expression in control or TK-co-cultured mDCTs with or without additional KCl at concentrations of 2 mM or 4 mM. GAPDH was used as a loading control. Data are representative of n=3-5 in each group.

Supplementary Figure 17 (Mu)

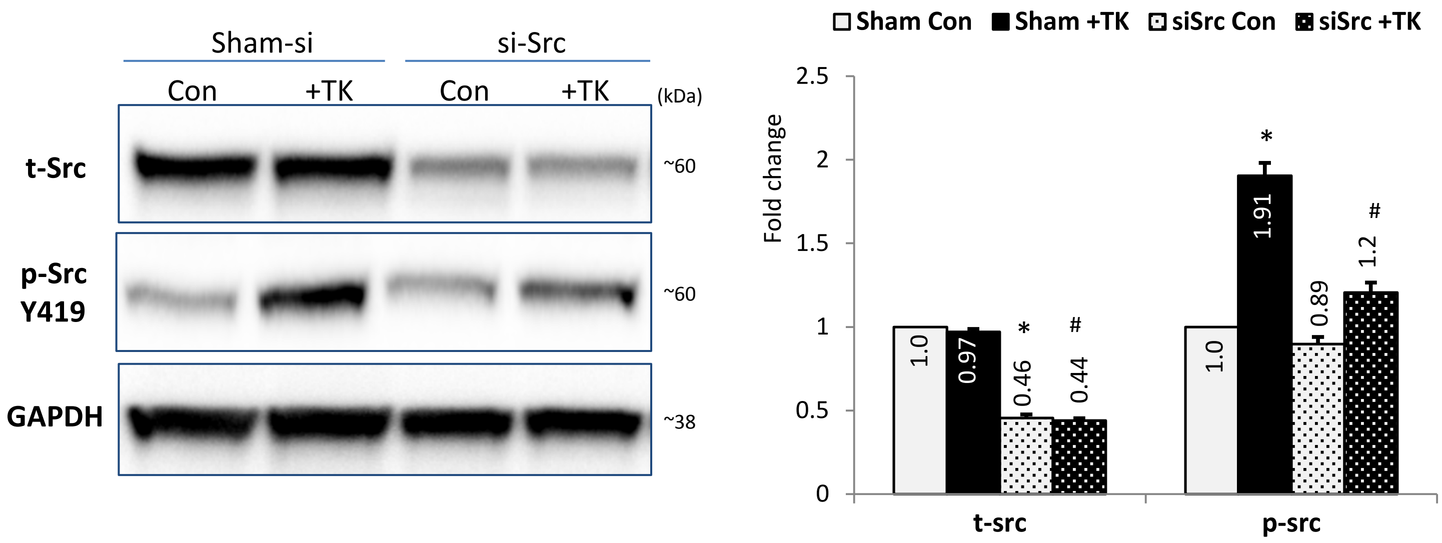

Supplementary Figure 17. Effects of siSrc on Src expression and activation in mDCTs with or without TK treatment. Although knockdown of Src did not dramatically decrease the active form of Src p-SrcY419 in control cells, it greatly prevented TK-induced increase of Src activation. GAPDH was used as a loading control. n=4 in each group. Data are means ± s.e. *p<0.01 vs. Control; #p<0.01 vs. sham+TK (ANOVA).

Supplementary Figure 18 (Mu)

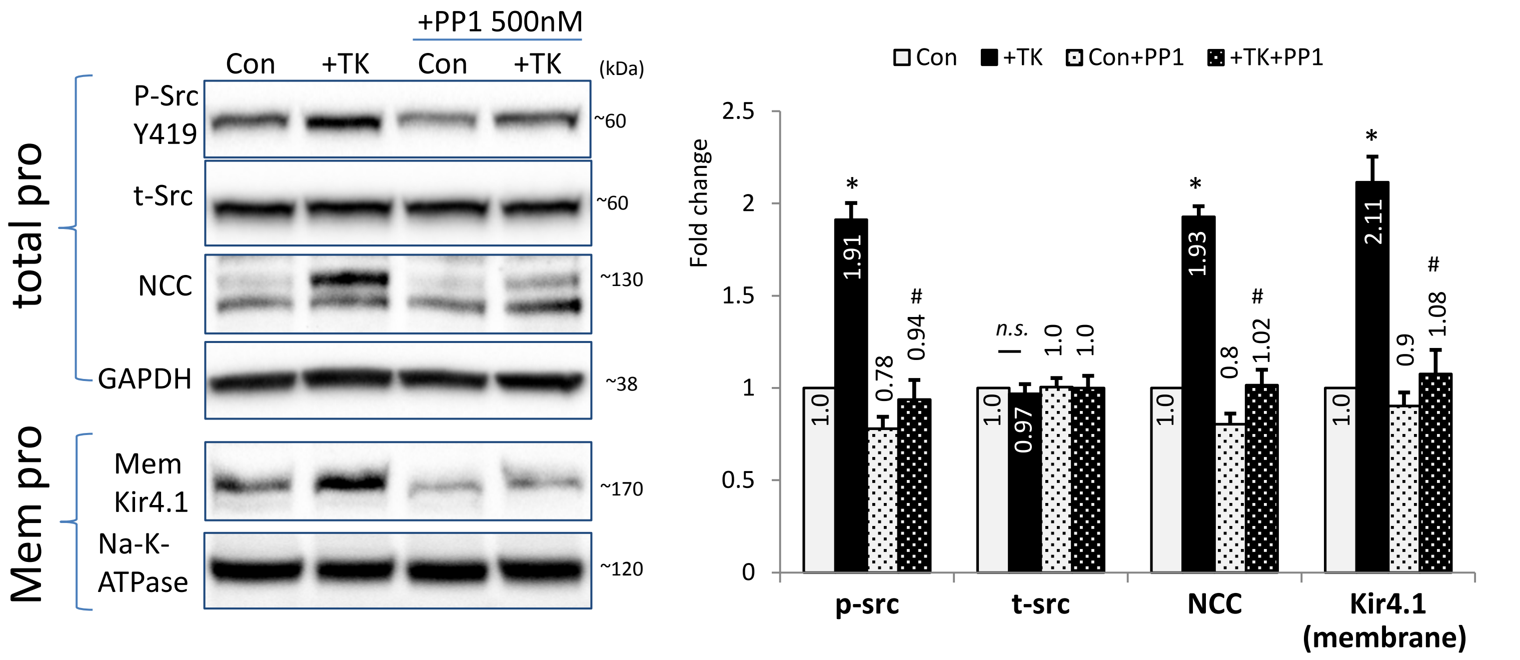

Supplementary Figure 18. Effects of Src inhibitor PP1 on TK-induced activation of Src, increase of membrane Kir4.1 and up-regulation of NCC in mDCTs. Na-K-ATPase or GAPDH were used as loading controls for membrane protein or total protein, respectively. n=3-4 in each group. Data are means ± s.e. *p<0.01 vs. Control; #p<0.01 vs. +TK (ANOVA).

Supplementary Figure 19 (Mu)

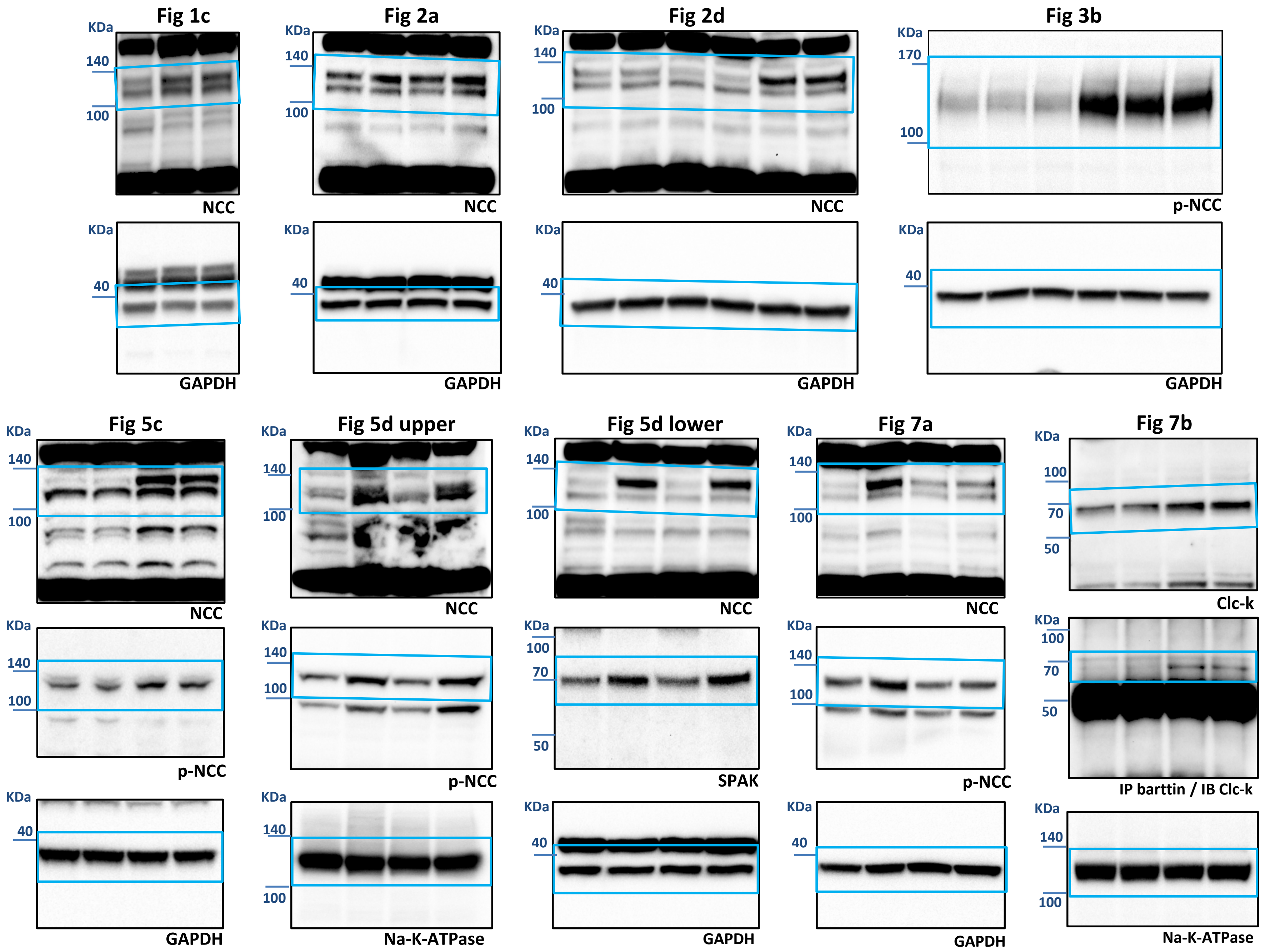

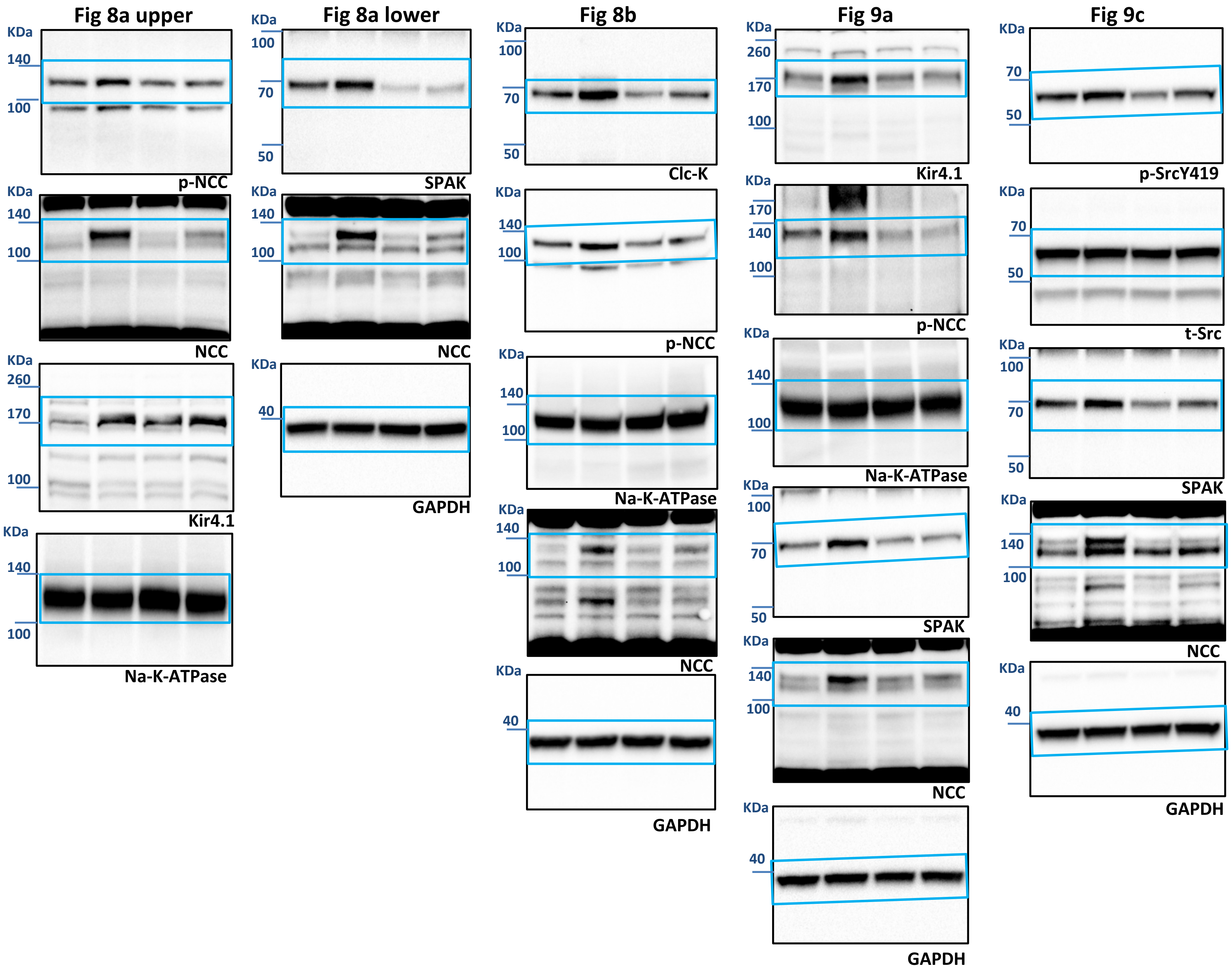

Fig 8a upper
KDa
140
100
p-NCC
KDa
140
100
NCC
KDa
260
170
100
Kir4.1
KDa
140
100
Na-K-ATPase
Fig 8a lower
KDa
100
70
50
SPAK
KDa
140
100
NCC
KDa
40
GAPDH
Fig 8b
KDa
100
70
50
Clc-K
KDa
140
100
p-NCC
KDa
140
100
Na-K-ATPase
KDa
140
100
NCC
KDa
40
GAPDH
Fig 9a
KDa
260
170
100
Kir4.1
KDa
170
140
100
p-NCC
KDa
140
100
Na-K-ATPase
KDa
100
70
50
SPAK
KDa
140
100
NCC
KDa
40
GAPDH
Fig 9c
KDa
70
50
p-SrcY419
KDa
70
50
t-Src
KDa
100
70
50
SPAK
KDa
140
100
NCC
KDa
40
GAPDH

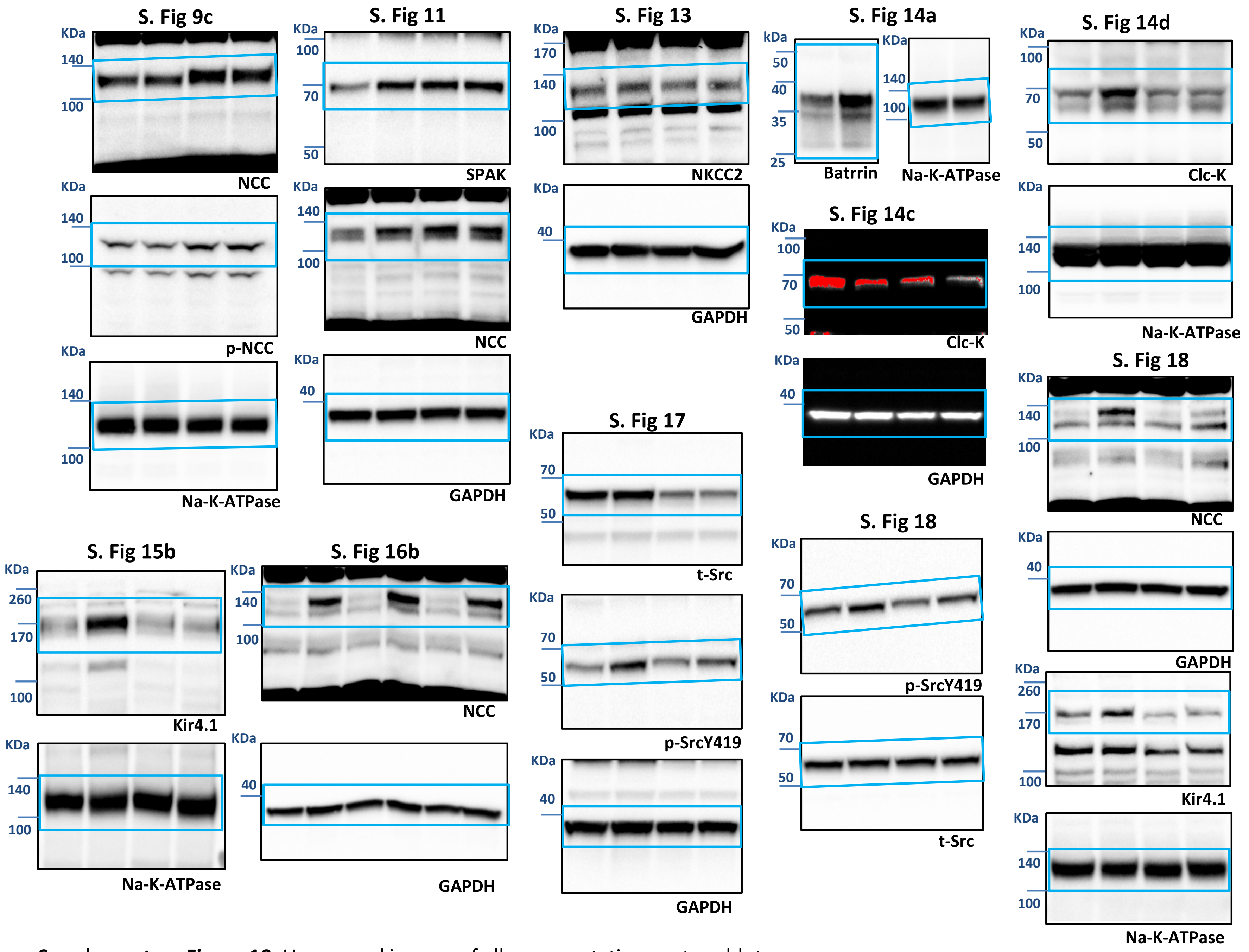

Supplementary Figure 19 Uncropped images of all representative western blots.

Supplementary Table 1 (Mu)

	Primary Antibodies							Secondary Antibodies			
	Antibody Target	Apparent position	Antibody supplier	Antibody Cat# /lot#	Antibody host	Antibody dilution	Incubation time	Antibody host/target	Antibody supplier	Antibody dilution	incubation time
Western Blot	NCC	~130/110 KDa	Abcam	ab95302/ GR89090	Rb (P)	1:1500	O/N	GaRb	Jackson immuno	1:10000	2h
	GAPDH	~38 KDa	Millipore	MAB374	Ms (M)	1:1000	O/N	GaMs	Jackson immuno	1:10000	2h
	p-NCC	~130 KDa	Ellison (OHSU)	#39,#40	Rb (P)	1:2000	O/N	GaRb	Jackson immuno	1:10000	2h
	Na-K-ATPase	~120 KDa	Abcam	ab76020	Rb (M)	1:75000	O/N	GaRb	Jackson immuno	1:10000	2h
	SPAK	~70 KDa	CST	#2281	Rb (P)	1:1000	O/N	GaRb	Jackson immuno	1:10000	2h
	Clc-K	~68 KDa	Alomone	ACL-004	Rb (P)	1:400	O/N	GaRb	Jackson immuno	1:10000	2h
	Kir4.1	~170 KDa (tetramer)	Alomone	APC-035	Rb (P)	1:600	O/N	GaRb	Jackson immuno	1:10000	2h
	p-Src Y419	~60KDa	Abcam	ab185617	Rb (M)	1:5000	O/N	GaRb	Jackson immuno	1:10000	2h
	t-Src	~60KDa	Abcam	ab109381	Rb (M)	1:15000	O/N	GaRb	Jackson immuno	1:10000	2h
	NKCC2	125KDa	Abcam	ab171747	Rb (M)	1:2000	O/N	GaRb	Jackson immuno	1:10000	2h
	Barttin	37KDa	SantaCruz	sc-365161	Ms (M)	1:500	O/N	GaMs	Jackson immuno	1:10000	2h
Immuno-staining	CD3	staining	Abcam	ab16669	Rb (M)	1:100	O/N	GaRb	Vector kit	1:2000	2h
	NCC	staining	Abcam	ab95302	Rb (P)	1:100	O/N	GaRb	Abcam 150077	1:200	2h
	CD8	staining	Novus	NBP2-12183	Rt (M)	1:100	O/N	GaRt	Abcam 150168	1:200	2h
	CD8	staining	Biolegend	100758	Rt (M)	1:100	O/N	N/A (primary labeled with Alexa 594)			
	Na-K-ATPase	Staining	Abcam	ab198367	Rb (M)	1:100	O/N	N/A (primary labeled with Alexa 647)			
	Rb=rabbit; Ms=mouse; Rt=Rat; (P)=polyclonal; (M)=monoclonal; GaRb=goat anti rabbit; GaMs=goat anti-mouse; GaRt=goat anti-rat; NFM=non-fatty milk; O/N=overnight										

Supplementary Table 1 Information of all antibodies for western blot or immuno-staining experiments.
